# Supplementary material for: Unsupervised representation learning on high-dimensional clinical data improves genomic discovery and prediction
Source: Nat Genet. 2024 Jul 8;56(8):1604–13. doi: 10.1038/s41588-024-01831-6 (PMC11319202; doi:10.1038/s41588-024-01831-6)
Supplement: Supplementary file 1 — Supplementary Notes, Supplementary Figs. 1–34 and Supplementary Tables 1–34. [file 41588_2024_1831_MOESM1_ESM.pdf]

nature genetics

Supplementary information https://doi.org/10.1038/s41588-024-01831-6

Unsupervised representation learning on high-dimensional clinical data improves genomic discovery and prediction

In the format provided by the authors and unedited

Contents

Supplementary Notes 2

(R)SPINCs reconstruct spirograms with few latent dimensions . . . 2

(R)SPINCs are consistent across random weight initializations . . . 2

REGLE embeddings encode information beyond EDFs . . . 2

(R)SPINCs GWAS . . . 3

PLENCs GWAS . . . 4

S-LDSC analyses of REGLE embeddings . . . 4

(R)SPINCs hits and loci compared to previous GWAS . . . 4

Functional significance of discovered loci . . . 5

(R)SPINCs are associated with overall survival . . . 5

SPINCs model architecture . . . 8

RSPINCs model architecture . . . 10

PLENCs model architecture . . . 11

RPLENCs model architecture . . . 12

Dataset acknowledgment . . . 15

Supplementary Figures 19

Supplementary Tables 44

Supplementary References 60

Supplementary Notes

(R)SPINCs reconstruct spirograms with few latent dimensions

The quality of the reconstruction from our models improves as we increase the latent dimension (i.e. the number of coordinates in SPINCs or RSPINCs) (Fig. 2b) and we observed highly accurate reconstruction of the input spirograms with just five SPINCs (Fig. 2c), identical to the number of EDFs we consider. In comparison, spirogram reconstructions from PCA with five PCs resulted in a $2\times$ higher reconstruction error, which indicates SPINCs and RSPINCs encoded more information about spirograms than PCs with the same number of coordinates (Fig. 2b). To have a fair comparison, SPINCs model and PCA were trained and evaluated on the same set of datasets. We observed a similar pattern of reconstruction errors using RSPINCs (Extended Data Fig. 2), though note that they are not directly comparable due to the inclusion of EDFs.

(R)SPINCs are consistent across random weight initializations

The objective function used to train deep learning models is typically not convex, so training runs with different initialization of weights can converge to substantially different local minima. To assess the stability of our encodings to unimportant changes in training, we generated five sets of SPINCs (dim=5) using different random seeds for initialization of weights, and computed Pearson correlation of the coordinates of each set with the coordinates of all other sets. Up to a change of signs and a permutation of the coordinates, which have no significance in our model or its downstream applications, we observed that the learned encodings are highly consistent (Extended Data Fig. 3).

REGLE embeddings encode information beyond EDFs

Some coordinates of SPINCs are highly correlated with known EDFs (Extended Data Fig. 5). For example, the 3rd coordinate of SPINCs is 96% correlated with FVC and 94% correlated with FEV_1, while the 2nd coordinate is 73% correlated with FEV_1/FVC (after flipping the signs) (Extended Data Fig. 5). Both RSPINCs coordinates have low correlation ($|R| < 0.3$) with all EDFs, which is expected since they were encouraged to learn only residual signals not captured by the EDFs (Extended Data Fig. 5). (R)SPINCs are also correlated with other predictors of lung function ("covariates"), such as age, sex, height, body mass index, and smoking status (Extended Data Fig. 5). To investigate if

(R)SPINCs include information beyond EDFs and covariates, we residualized both the EDFs and the covariates from (R)SPINCs and computed correlation with tabular UK Biobank features. Multiple groups of fields strongly and significantly correlated with the (R)SPINCS even after residualizing the EDFs and the covariates, including asthma, breathing issues, cognitive function, and allergies (Supplementary Tables 4 to 6).

We observed similar qualitative results for (R)PLENCs, which also show strong correlation with known PPG EDFs (Extended Data Fig. 6). For example, the 3rd coordinate of PLENCs is 75% correlated with the position of the shoulder and 74% correlated with the position of the peak, while the first coordinate is 58% correlated with the pulse rate (after sign flip). PLENCs also show correlation with other predictors of circulatory function (Extended Data Fig. 6). After residualizing EDFs and covariates from PLENCs, we still observed multiple groups of fields strongly and significantly correlated with the residualized PLENCs coordinates, including pulse rate, systolic and diastolic blood pressure, and position of shoulder (Supplementary Tables 7 and 8).

Finally, using the Cox proportional hazards model, we observed that both (R)SPINCs and (R)PLENCs are associated with overall survival. For example, the third coordinate of SPINCs had a hazard ratio of 0.68 (95% CI, 0.65 to 0.71; P=1.6E-83) implying the hazard of death decreased by 32% per one standard deviation increase in the coordinate (Supplementary Notes, Extended Data Fig. 7, Supplementary Figs. 1 to 3, Supplementary Table 9, Methods). SPINC hazard ratios are similar to FVC and slightly worse than FEV_1 (0.64 vs 0.68; Supplementary Table 9), given that FEV_1 is a known clinical feature for COPD, REGLE can extract important clinical features from HDCD.

(R)SPINCs GWAS

We generated SPINCs (dim=5) and RSPINCs (dim=2, in addition to 5 EDFs) for all individuals with valid first-visit spirograms in UK Biobank (Extended Data Fig. 1, Supplementary Figs. 4 and 5; Methods). Then, we performed GWAS on all European GIA (genetically inferred ancestry) individuals (n=324,702) on all encoding coordinates and 5 EDFs using BOLT-LMM [1, 2], adjusting for age, sex, age^2, age $\times$ sex, height, height^2, body mass index, smoking status, pack-years of smoking, the type of genotyping array, and the top 15 genetic principal components (Methods). The Manhattan plots of 5 SPINCs and 2 RSPINCs GWAS are illustrated in Supplementary Figs. 6 to 10 and Supplementary Figs. 11 and 12, respectively. The intercept term from the stratified linkage disequilibrium score regression (S-LDSC) [3] was close to 1 (Supplementary Table 10) for the GWAS of SPINCs and RSPINCs, indicating minimal confounding bias. The SNP-heritability estimated from

S-LDSC for SPINCs and RSPINCs showed strong genetic components (Supplementary Table 10). For comparison, we also performed GWAS on the first 5 PCs of the raw spirograms following the same steps.

PLENCs GWAS

We generated PLENCs (dim=5) for all individuals with valid first-visit PPG in UK Biobank (Methods). Then, we performed GWAS on all European-GIA individuals (n=141,275) on all encoding coordinates and 5 EDFs using BOLT-LMM [1, 2], adjusting for age, sex, age^2, age $\times$ sex, height, height^2, body mass index, the type of genotyping array, and the top 15 genetic principal components (Methods). The Manhattan plots of 5 PLENCs GWAS are illustrated in Supplementary Figs. 13 to 17. The intercept term from the stratified linkage disequilibrium score regression (S-LDSC) [3] was close to 1 (Supplementary Table 10) for the GWAS of PLENCs, indicating minimal confounding bias. The SNP-heritability estimated from S-LDSC for PLENCs showed strong genetic components (Supplementary Table 10). For comparison, we also performed GWAS on the first 5 PCs of the raw PPG following the same steps.

S-LDSC analyses of REGLE embeddings

S-LDSC analysis demonstrates that each of the SPINCs, RSPINCs, PLENCs, and RPLENCs have significant SNP heritability (Supplementary Table 10). For example, the SNP heritability of SPINCs ranged from 4.3% for SPINC$_5$ to 24.8% for SPINC$_3$. EDFs maximum heritability and SPINCs are not significantly different while in the case of PPG maximum PLENCs heritability is significantly larger than EDFs (0.13 (0.01) vs 0.07 (0.01)). As expected EDFs heritability are very similar as these phenotypes are highly correlated. Importantly, the RSPINCs, which are relatively uncorrelated with the EDFs, were also significantly heritable (4.5-16.2%), indicating the existence of additional heritable signals within the spirograms (Supplementary Table 10).

(R)SPINCs hits and loci compared to previous GWAS

GWAS on 5 SPINCs detected 575 independent genome-wide significant (GWS) loci ($R^2 \leq 0.1$ and $P \leq 5 \times 10^{-8}$) after merging hits within 250kb together. To compare our results to known lung function loci from previous literature, we combined the largest published GWAS on lung function (using FEV$_1$, FVC, PEF, and FEV$_1$/FVC) from [4] (580,869 individuals, compared to our

324,702 individuals in UKB) with all lung function-related loci in the NHGRI-EBI GWAS Catalog [5] (Methods). This resulted in 1104 independent loci after merging loci by distance (250kb), hereafter referred to as "previously known loci". Most GWS loci from SPINCs and EDFs+RSPINCs recover previously known loci (89% for SPINCs, 90% for EDFs+RSPINCs). Out of 575 GWS SPINCs loci, 65 (11%) were not previously known, compared to 32 from EDFs and 15 from PCA. Of 659 EDFs+RSPINCs GWS loci, 63 (10%) were not previously known (Table 1).

Functional significance of discovered loci

We ran GREAT and GARFIELD for functional enrichment. For GREAT, we combined GWS loci from SPINCs GWAS by merging those within 250 kb of each other and analyzed the resulting set of independent regions (n=575), and performed the analogous analysis for EDFs (n=613) and EDFs+RSPINCs (n=659). The strongest consistent enrichments were Gene Ontology terms related to development and morphogenesis (Supplementary Table 34). Notably, significant enrichments for EDFs were largely found to be even more significantly enriched in the EDFs+RSPINCs, consistent with the RSPINCs identifying additional genes influencing the same biological pathways as EDFs (Extended Data Fig. 8, $P = 2.4 \times 10^{-25}$, two-sided paired t-test).

In addition, using GARFIELD to test the enrichment of SPINCs and RSPINCs GWAS with DNase I hypersensitive hotspots, we observed a strong enrichment of SPINCs and RSPINCs in fetal lung (Supplementary Figs. 20 to 24 and Supplementary Figs. 25 and 26). Notably, we found a strong enrichment for RSPINC_2 in blood (Supplementary Fig. 26).

(R)SPINCs are associated with overall survival

We performed survival analysis for European GIA individuals in the validation set ($n = 65,266$) across EDFs, SPINCs, and RSPINCs, fitting a Cox proportional hazards regression model to UKB death registry data while controlling for age and sex as covariates (Methods and Supplementary Table 9). The EDF and SPINC_3 hazard ratios (HR) for all cause mortality (i.e., overall survival) were 0.640 (95% CI $[0.615, 0.666]$), 0.679 ($[0.653, 0.77]$), 0.685 ($[0.656, 0.715]$), 0.689 ($[0.663, 0.716]$), 0.752 ($[0.728, 0.777]$), and 0.806 ($[0.786, 0.826]$) per one standard deviation increase in FEV_1, SPINC_3, FVC, $\text{FEF}_{25\text{-}75\%}$, PEF, and FEV_1/FVC, respectively, suggesting that these features are strongly to moderately associated with improved survival or longer time-to-death. Conversely, the RSPINC_2, SPINC_1, SPINC_2, and SPINC_4 HRs of 1.064 (95% CI $[1.032, 1.097]$), 1.078 ($[1.045, 1.111]$), 1.086

($[1.052, 1.121]$), and 1.135 ($[1.101, 1.169]$) per standard deviation increase suggesting that these features are moderately associated with shorter time-to-death. Note that the SPINC_1 model fails the proportional-hazards (PH) assumption ($p = 0.013$). Thus, the estimated hazard ratio is time-varying, and the reported value should be interpreted as giving the net direction of association. Neither SPINC_5 nor RSPINC_1 are significantly associated with survival. Kaplan-Meier curves for overall survival (OS) stratified by feature indicate that OS declines more rapidly for patients with higher SPINC_1 and lower SPINC_3 scores (Extended Data Fig. 7, Supplementary Fig. 1).

(R)SPINCs PRS transferred to COPDGene

For the COPDGene dataset, we computed PRS of all individuals using the same variant effect sizes obtained in UKB and the same linear weights to combine the PRS as before after matching variants. We used the "race" field in COPDGene as a proxy for genetic ancestry and computed PRS performance in the two available subsets separately: "Non-Hispanic White" and "African American". We observed that for COPD, the SPINCs COPD PRS outperforms the EDFs COPD PRS for both subset of individuals in COPDGene for all four evaluation metrics (AUC-ROC, AUC-PR, top-decile prevalence, and Pearson correlation). In the "Non-Hispanic White" subset (n=6,576), which matches the UKB ancestry group on which the PRS was trained, all four metrics are statistically significant (paired bootstrapping; Fig. 5a, Supplementary Table 21). In the "African American" subset (n=3,140), differences were statistically significant for AUC-ROC and Pearson correlation (Supplementary Table 21). The EDFs+RSPINCs COPD PRS significantly outperformed the EDFs COPD PRS in "Non-Hispanic White" in AUC-ROC and Pearson correlation, but did not in the "African American" subset (Supplementary Table 21).

High association between REGLE encoding PRSs and UKB phenotype PRSs

To assess the influence of (R)SPINCs and PLENCs on traits and health outcomes, we performed phenome-wide association studies (PheWAS). We compared pruning+thresholding PRSs of all (R)SPINCs and PLENCs coordinates to PRSs of 7,145 phenotypes computed by the Pan-UK Biobank consortium (Methods, `https://pan.ukbb.broadinstitute.org`). For SPINCs, significant associations with strong correlation magnitude are driven by the 2nd and 3rd coordinates, which as mentioned above are strongly correlated with EDFs, and include expected phenotypes such as FEV_1, FEV_1/FVC, and PEF (Supplementary Tables 25 and 26). These coordinates also show strong corre-

lation to other diseases and traits associated with alterations in lung function, identifying relationships with systemic lupus erythematosus [6, 7], thyroid dysfunction [8], and gluten-free diet [9]. For RSPINCs, significant correlations with strong magnitude are nearly all driven by RSPINC_1 and also include the same diseases (Supplementary Tables 27 and 28). For (R)PLENCs, we observed all coordinates show significant correlation with different traits including blood traits (red blood cell, eosinophil count, age high blood pressure diagnosed, and hemoglobin concentration), PPG traits (pulse rate and pulse wave reflection index), ECG traits (QRS duration and P duration), blood pressure, and vascular/heart problems (Supplementary Tables 29 to 32). The strongest correlation was obtained from 1st PLENCs coordinate with pulse rate (R=-0.67; P $\leq$ 1.00E-300) and ECG heart rate (R=-0.52; P $\leq$ 1.00E-300) and 1st RPLENCs coordinate with Pulse wave peak to peak time (R=-0.31; P $\leq$ 1.00E-300).

REGLE differences with conditional VAE

Conceptually, REGLE is closely related to conditional VAE [10], a conditional generative model created to enable class-conditioned sample generation. The main difference between our approach and conditional VAE is that our encoder neither takes EDFs as input nor uses a conditional prior network. As a result, our trained encoder can generate residual encodings directly from input HDCD without using EDFs as an additional input.

Advantages of VAE over Traditional Autoencoder

First, due to the usage of factorized Gaussian prior distribution in VAEs, the coordinates of the latent representation are minimally correlated (Extended Data Fig. 5), which increases the combined power of the downstream GWASs. As a result, the PRSs of the learned encoding have lower correlation and contain relatively orthogonal genetic signals compared to EDFs (Supplementary Table 33), which may contribute to the superior accuracy of the disease/trait specific PRS created by the REGLE framework. Second, the learned representations are stable up to changes in sign or order as we observed empirically (Extended Data Fig. 3), potentially due to a grounding effect of a VAE prior in the probabilistic model. As changes in sign or order do not affect genetic discovery–though they may affect the sign of the effect size–the results of the REGLE framework are stable and replicable. Regular autoencoders without a prior do not have this stability property as they can learn any invertible linear transformation of a specific learned representation.

Futures model architecture and training strategies

Some ideas which may warrant further investigation include: 1) using previously proposed modifications to the VAE loss function and the training procedure to maximize the degree of disentanglement of coordinates while balancing the reconstruction error [11–13], 2) incorporating an additional loss term to explicitly discourage correlation between RSPINCs and EDFs, and 3) introducing (semi-) supervision in model training to overcome the limitations of purely unsupervised training [14].

Summary statistics conditional and joint analysis

We applied conditional and joint analysis (COJO) on a set of previously known loci using GCTA (genome-wide complex trait analysis) software (version `1.93.3beta`) and we set `-cojo-cond` to the set of known loci. We provided 10,000 unrelated European GIA samples randomly chosen from UKB as the reference samples, which is the same reference used to perform LD clumping to define hits, as part of GCTA-COJO input parameters.

SPINCs model architecture

```
Encoder:
==============================================================
 Layer (type)                Output Shape         Param #
==============================================================
 vae_encoder_input (InputLayer) [(None, 1000, 2)]    0
 conv1d (Conv1D)              (None, 1000, 8)      168
 max_pooling1d (MaxPooling1D)  (None, 500, 8)       0
 conv1d_1 (Conv1D)            (None, 500, 16)      1296
 max_pooling1d_1 (MaxPooling1D) (None, 250, 16)      0
 conv1d_2 (Conv1D)            (None, 250, 32)      5152
 max_pooling1d_2 (MaxPooling1D) (None, 125, 32)      0
 flatten (Flatten)            (None, 4000)         0
 dense (Dense)                (None, 64)           256064
 dense_1 (Dense)              (None, 64)           4160
 dense_2 (Dense)              (None, 64)           4160
```

```
 z_mean (Dense)              (None, 5)            325

 z_log_var (Dense)           (None, 5)            325

 gaussian_sampling           (None, 5)            0
 (GaussianSampling)
==============================================================
Total params: 271,650
Trainable params: 271,650

Decoder:
=================================================================
 Layer (type)                Output Shape              Param #
=================================================================
 vae_decoder_input           [(None, 5)]               0
 (InputLayer)

 dense_3 (Dense)             (None, 64)                384

 dense_4 (Dense)             (None, 64)                4160

 dense_5 (Dense)             (None, 64)                4160

 dense_6 (Dense)             (None, 4000)              260000

 reshape (Reshape)           (None, 125, 32)           0

 up_sampling1d (UpSampling1D) (None, 250, 32)          0

 conv1d_transpose            (None, 250, 16)           5136
 (Conv1DTranspose)

 up_sampling1d_1             (None, 500, 16)           0
 (UpSampling1D)

 conv1d_transpose_1          (None, 500, 8)            1288
 (Conv1DTranspose)

 up_sampling1d_2             (None, 1000, 8)           0
 (UpSampling1D)

 conv1d_transpose_2          (None, 1000, 2)           162
 (Conv1DTranspose)

=================================================================
Total params: 275,290
```

```
Trainable params: 275,290
```

RSPINCs model architecture

```
Encoder:
=================================================================
 Layer (type)                Output Shape              Param #
=================================================================
 vae_encoder_input (InputLayer) [(None, 1000, 1)]      0
 conv1d (Conv1D)             (None, 1000, 8)           88
 max_pooling1d (MaxPooling1D)   (None, 500, 8)         0
 conv1d_1 (Conv1D)           (None, 500, 16)           1296
 max_pooling1d_1 (MaxPooling1D) (None, 250, 16)        0
 conv1d_2 (Conv1D)           (None, 250, 32)           5152
 max_pooling1d_2 (MaxPooling1D) (None, 125, 32)        0
 flatten (Flatten)           (None, 4000)              0
 dense (Dense)               (None, 64)                256064
 dense_1 (Dense)             (None, 64)                4160
 dense_2 (Dense)             (None, 64)                4160
 z_mean (Dense)              (None, 2)                 130
 z_log_var (Dense)           (None, 2)                 130
 gaussian_sampling           (None, 2)                 0
 (GaussianSampling)
=================================================================
Total params: 271,180
Trainable params: 271,180


Concatenate (inject 5 EDFs into encoder output):
=================================================================
 Layer (type)                Output Shape              Param #
=================================================================
 concatenate (Concatenate)   (None, 7)                 0
```

Decoder:

```
=================================================================
 Layer (type)                Output Shape              Param #
=================================================================
 vae_decoder_input           [(None, 7)]              0
 (InputLayer)
 dense_3 (Dense)             (None, 64)                512
 dense_4 (Dense)             (None, 64)                4160
 dense_5 (Dense)             (None, 64)                4160
 dense_6 (Dense)             (None, 4000)              260000
 reshape (Reshape)           (None, 125, 32)           0
 up_sampling1d               (None, 250, 32)           0
 (UpSampling1D)
 conv1d_transpose            (None, 250, 16)           5136
 (Conv1DTranspose)
 up_sampling1d_1             (None, 500, 16)           0
 (UpSampling1D)
 conv1d_transpose_1          (None, 500, 8)            1288
 (Conv1DTranspose)
 up_sampling1d_2             (None, 1000, 8)           0
 (UpSampling1D)
 conv1d_transpose_2          (None, 1000, 1)           81
 (Conv1DTranspose)
=================================================================
Total params: 275,337
Trainable params: 275,337
```

PLENCs model architecture

Encoder:

```
=================================================================
 vae_encoder_input (InputLayer)  [(None, 100, 1)]       0
 conv1d (Conv1D)           (None, 100, 8)            88
```

```
 max_pooling1d (MaxPooling1D)  (None, 50, 8)          0

 conv1d_1 (Conv1D)         (None, 50, 16)           1296

 max_pooling1d_1 (MaxPooling1D)  (None, 25, 16)         0

 flatten (Flatten)         (None, 400)              0

 dense (Dense)             (None, 64)               25664

 dense_1 (Dense)           (None, 64)               4160

 dense_2 (Dense)           (None, 64)               4160

 z_mean (Dense)            (None, 5)                325

 z_log_var (Dense)         (None, 5)                325

 gaussian_sampling (Gaussian Sampling)  (None, 5)              0
=================================================================


Decoder:
=================================================================
 vae_decoder_input (InputLayer)  [(None, 5)]            0

 dense_3 (Dense)           (None, 64)               384

 dense_4 (Dense)           (None, 64)               4160

 dense_5 (Dense)           (None, 64)               4160

 dense_6 (Dense)           (None, 400)              26000

 reshape (Reshape)         (None, 25, 16)           0

 up_sampling1d (UpSampling1D)  (None, 50, 16)         0

 conv1d_transpose (Conv1DTranspose)  (None, 50, 8)          1288

 up_sampling1d_1 (UpSampling1D)  (None, 100, 8)         0

 conv1d_transpose_1 (Conv1DTranspose)  (None, 100, 1)         81
=================================================================


Total params: 72,091

Trainable params: 72,091
```

RPLENCs model architecture

```
Encoder (34948 params):
=================================================================
```

```
 Layer (type)                Output Shape              Param #
=================================================================
vae_encoder_input (InputL  [(None, 100, 1)]          0
ayer)
conv1d (Conv1D)            (None, 100, 8)            48
max_pooling1d (MaxPooling  (None, 50, 8)             0
1D)
conv1d_1 (Conv1D)          (None, 50, 16)            656
max_pooling1d_1 (MaxPooli  (None, 25, 16)            0
ng1D)
flatten (Flatten)          (None, 400)               0
dense (Dense)              (None, 64)                25664
dense_1 (Dense)            (None, 64)                4160
dense_2 (Dense)            (None, 64)                4160
z_mean (Dense)             (None, 2)                 130
z_log_var (Dense)          (None, 2)                 130
gaussian_sampling (Gaussi  (None, 2)                 0
anSampling)
=================================================================

Concatenate (inject 5 EDFs into encoder output):
=================================================================
 Layer (type)                Output Shape              Param #
=================================================================
concatenate (Concatenate)   (None, 7)                 0
=================================================================

Decoder (35521 params):
=================================================================
 Layer (type)                Output Shape              Param #
=================================================================
vae_decoder_input (InputL  [(None, 7)]               0
```

```
ayer)
dense_3 (Dense)            (None, 64)                512
dense_4 (Dense)            (None, 64)                4160
dense_5 (Dense)            (None, 64)                4160
dense_6 (Dense)            (None, 400)               26000
reshape (Reshape)          (None, 25, 16)            0
up_sampling1d (UpSampling  (None, 50, 16)            0
1D)
conv1d_transpose (Conv1DT  (None, 50, 8)             648
ranspose)
up_sampling1d_1 (UpSampli  (None, 100, 8)            0
ng1D)
conv1d_transpose_1 (Conv1  (None, 100, 1)            41
DTranspose)
=================================================================
Total params: 70469
Trainable params: 70469
```

Dataset acknowledgment

Advarra IRB (Columbia, MD) waived ethical approval for this work involving de-identified medical imagery and metadata under 45 CFR 46. Work related to genomics data were additionally reviewed by the respective data sources: UK Biobank, COPDGene, eMERGE III, EPIC Norfolk, and Indiana Biobank.

UK Biobank dataset acknowledgment

This research has been conducted using the UK Biobank Resource under Application Number 65275.

COPDGene dataset acknowledgment

This research used data generated by the COPDGene study, which was supported by NIH grants U01 HL089856 and U01 HL089897. The COPDGene project is also supported by the COPD Foundation through contributions made by an Industry Advisory Board comprised of Pfizer, AstraZeneca, Boehringer Ingelheim, Novartis, and Sunovion.

EPIC Norfolk dataset acknowledgment

The EPIC-Norfolk study (DOI 10.22025/2019.10.105.00004) has received funding from the Medical Research Council (MR/N003284/1 MC-UU_12015/1 and MC_UU_00006/1) and Cancer Research UK (C864/A14136). The genetics work in the EPIC-Norfolk study was funded by the Medical Research Council (MC_PC_13048). We are grateful to all the participants who have been part of the project and to the many members of the study teams at the University of Cambridge who have enabled this research.

Indiana Biobank dataset acknowledgment

This study was made possible, in part, with support from the Indiana Clinical and Translational Sciences Institute funded, in part by Award Number UL1TR002529 from the National Institutes of Health, National Center for Advancing Translational Sciences, Clinical and Translational Sciences Award, and the National Center for Research Resources, Construction grant number RR020128 and the Lilly Endowment. The content is solely the responsibility of the authors and does not necessarily represent the official views of the National Institutes of Health. The authors acknowledge the

Indiana University Pervasive Technology Institute for providing [HPC (Big Red II, Karst, Carbonate), visualization, database, storage, or consulting] resources that have contributed to the research results reported within this paper.

eMERGE III dataset acknowledgment

This research used data generated by the eMERGE III study which was obtained from dbGaP under accession phs001584.v2.p2. See Supplementary Note for full acknowledgement for eMERGE III.

Cincinnati Children's Hospital Medical Center (CCHMC) – Acknowledgement Text: CCHMC is a participating pediatric institution for Phase III of the eMERGE network, a national consortium selected to expand best practices and knowledge in effective implementation of genomic medicine to pursue a broad-based program sufficiently large to define health outcomes associated with rare variants in ~100 clinically relevant genes. CCHMC Principal Investigators (PIs) have contributed sequencing data generated from the following cohorts: (1) Better Outcomes for Children (BOfC). Since January, 2011, the Cincinnati Biobank has managed the sample repository developed through the BOfC protocol (PI: John Harley), an institution-wide broad based consent project to utilize remnant clinical samples for biomedical research from participants consented at registration. This project is made possible by institutional resources. To date, over 261,000 participants have consented to BOfC and DNA samples are stored from more than 84,000 unique patients. Through an IRB approved protocol led by Dr. Bahram Namjou (2015-7778), 2,800 samples were selected for sequencing on the eMERGE sequencing panel representing >15 primary phenotypes including Arrhythmia, Asthma, Cardiomyopathy, Chronic kidney disease, Ehlers-Danlos Syndrome, Hyperlipidemia, Autistic behavior, and Tuberous Sclerosis 1. This project is made possible by the support of U01HG008666 (PI: John Harley). (2) Return of eMERGE III Genomic Results. Through an IRB approved protocol led by Dr. Melanie Myers (2016-3361), 200 adolescent patients and their parents were consented to examine (1) their choices about results to be returned on the eMERGE sequencing panel, (2) their responses to learning negative genetic test results, and (3) the parents' responses after learning their children's positive results. All 200 participants provided blood samples. Extracted DNA samples were sequenced on the eMERGE sequencing panel. Results are to be returned to participants. This project is made possible by the support of U01HG008666 (PI: John Harley). Patients of interest were identified using anthropometric measurements, clinical data and ICD codes extracted from the EPIC electronic medical record (EMR). The extraction of data from the EMR into the de-identified data warehouse, i2b2, was made possible by

institutional resources and UL1RR026314/UL1TR001425, the Cincinnati Center for Clinical and Translational Sciences and Training Grant (PI: James Heubi). Children's Hospital of Philadelphia (CHOP) Center for Applied Genomics, The Children's Hospital of Philadelphia Samples and associated genomic and phenotype data used in this study were provided by the Center for Applied Genomics at the Children's Hospital of Philadelphia (CHOP). Support for genotyping was provided by an Institutional Development Award from CHOP. Support for sequencing was provided by the National Institutes of Health through an award from the National Human Genome Research Institute's Electronic Medical Records and Genomics (eMERGE) program (U01HG008684). Columbia University Samples and data used in this study were provided by the Center for Glomerular Diseases at Columbia University, the Columbia Transplant Programs, the DataBase Shared Resource at the Herbert Irving Comprehensive Cancer Center, and the Institute for Genomic Medicine at Columbia University. Funding support for the Columbia eMERGE III research study was provided by a U01 grant from the National Human Genome Research Institute (U01HG008680; PIs – Chunhua Weng, PhD; George Hripcsak, MD; Ali Gharavi, MD). Geisinger Funding for the MyCode® sample and data collection was provided by grants from Commonwealth of Pennsylvania, the Clinic Research Fund of Geisinger Clinic, and the Regeneron Genetics Center. Partners Health Care (Harvard University) Samples and data used in this study were provided by the Partners Health Care Biobank (https://biobank.partners.org/). Funding support for the Partners Biobank was provided by Partners Health Care and Partners Personalized Medicine. Assistance with phenotype harmonization was provided by the eMERGE Coordinating Center (Grant number U01HG04603). Additional support was provided by the NIH, NHGRI eMERGE Network (U01HG 5U01HG008685-03). Funding support for genotyping, which was performed at the Translational Genomics Core, Partners Personalized Medicine and funded by Partners Personalized Medicine. Assistance with phenotype harmonization and genotype data cleaning was provided by the eMERGE Administrative Coordinating Center (U01HG004603) and the National Center for Biotechnology Information (NCBI). The datasets used for the analyses described in this manuscript were obtained from dbGaP at http://www.ncbi.nlm.nih.gov/gap through dbGaP accession number; phs000944.v1.p1. Kaiser Washington/University of Washington Funding support for Alzheimer's Disease Patient Registry (ADPR) and Adult Changes in Thought (ACT) study was provided by a U01 from the National Institute on Aging (Eric B. Larson, PI, U01AG006781). A gift from the 3M Corporation was used to expand the ACT cohort. DNA aliquots sufficient for GWAS from ADPR Probable AD cases, who had been enrolled in Genetic Differences in Alzheimer's Cases and Controls (Walter Kukull, PI,

R01 AG007584) and obtained under that grant, were made available to eMERGE without charge. Funding support for genotyping, which was performed at Johns Hopkins University, was provided by the NIH (U01HG004438). Genome-wide association analyses were supported through a Cooperative Agreement from the National Human Genome Research Institute, U01HG004610 (Eric B. Larson, PI). Assistance with phenotype harmonization and genotype data cleaning was provided by the eMERGE Administrative Coordinating Center (U01HG004603) and the National Center for Biotechnology Information (NCBI). The datasets used for the analyses described in this manuscript were obtained from dbGaP at http://www.ncbi.nlm.nih.gov/gap through dbGaP accession number phs000234.v1.p1. Mayo Clinic Samples and associated genotype and phenotype data used in this study were provided by the Mayo Clinic. Funding support for the Mayo Clinic was provided through a cooperative agreement with the National Human Genome Research Institute (NHGRI), Grant #: UOIHG004599, UOIHG006379; and the Mayo Center for Individualized Medicine. Funding support for sequencing, which was performed at The Baylor Human Genomics Sequencing Center, was provided by the NIH. Assistance with phenotype harmonization and genotype data cleaning was provided by the eMERGE Administrative Coordinating Center and the National Center for Biotechnology Information (NCBI). Northwestern University Samples and data used in this study were obtained from patients of Northwestern Medicine, Chicago, IL, who were recruited for the eMERGE II Pharmacogenomics Study and the eMERGE III Your Genes and Your Health Study. The Pharmacogenomics Study, a supplement to the Northwestern eMERGE II Project (U01HG006388) and the Your Genes and Your Health Study (U01HG008673) were funded through the NIH, NHGRI eMERGE Network. Vanderbilt University Funding support for the Vanderbilt Genome-Electronic Records (VGER) project was provided through a cooperative agreement (U01HG008672) with the National Human Genome Research Institute (NHGRI) with additional funding from the National Institute of General Medical Sciences (NIGMS). The dataset(s) used for the analyses described were obtained from Vanderbilt University Medical Center. Assistance with phenotype harmonization and genotype data cleaning was provided by the eMERGE Administrative Coordinating Center (U01HG004603) and the National Center for Biotechnology Information (NCBI). The datasets used for the analyses described in this manuscript were obtained from dbGaP at http://www.ncbi.nlm.nih.gov/gap through dbGaP accession number phs000188.v1.p1.

Supplementary Figures

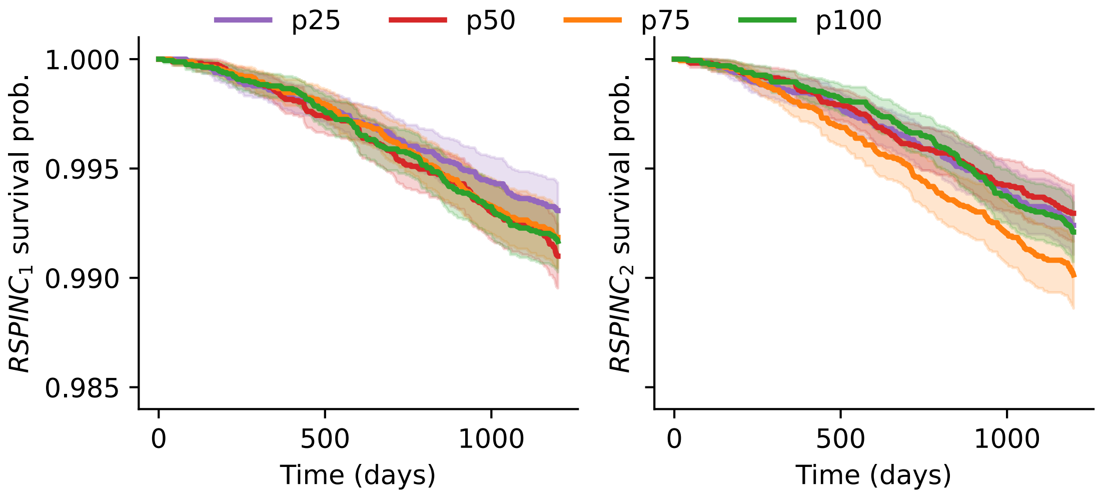

Supplementary Fig. 1: **RSPINCs Kaplan-Meier curves**. Kaplan-Meier curves estimating the overall survival (OS) function for European GIA individuals in the validation dataset (n=65,266). Individuals were stratified into quartiles using each RSPINC coordinate (e.g., "p25" denotes the bottom quartile) and OS curves were constructed using the standard Kaplan-Meier estimator with bootstrapping. The center lines are the means and the error bands are boostrapped 95% confidence intervals. See Supplementary Table 9 for the corresponding hazard ratios per standard deviation.

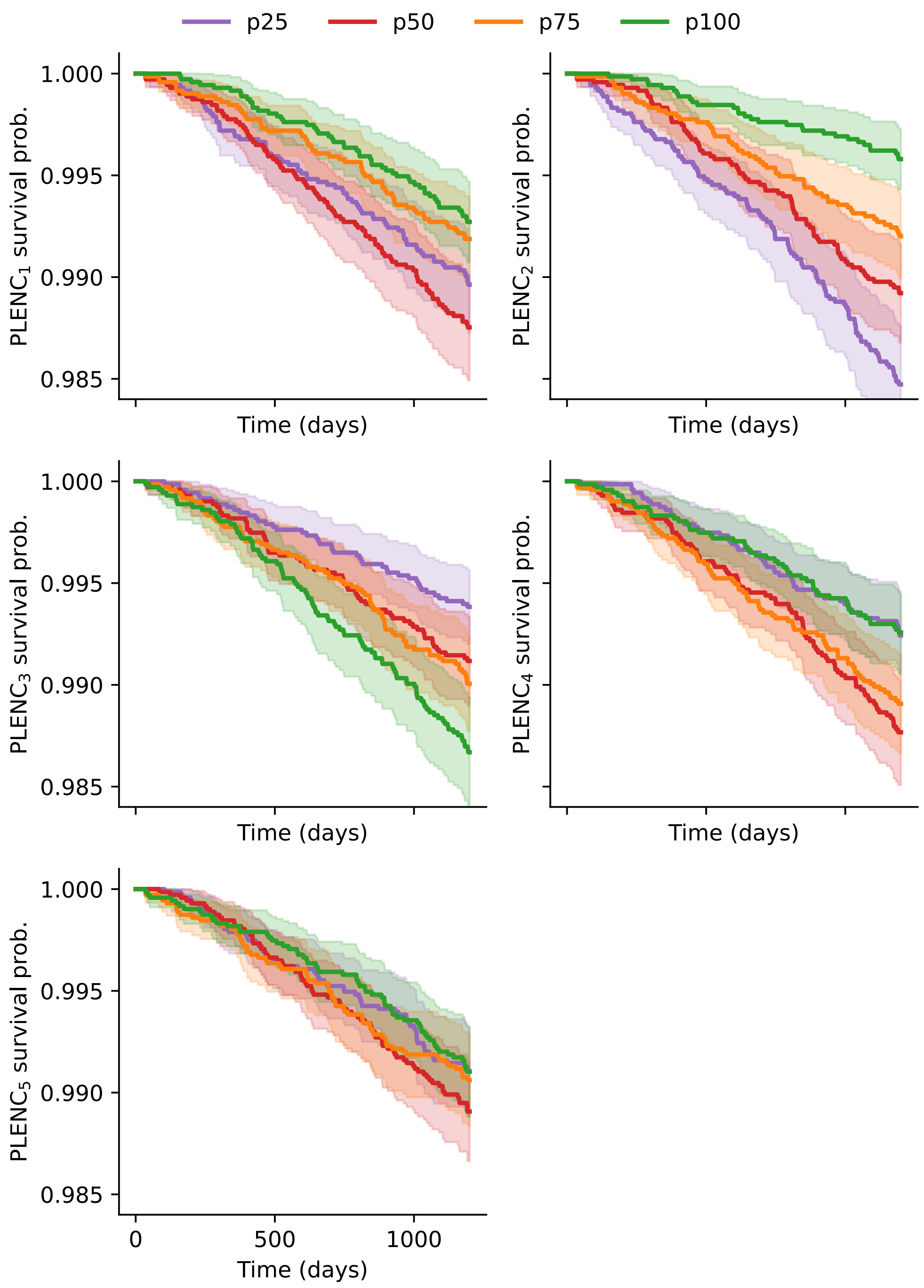

Supplementary Fig. 2: **PLENCs Kaplan-Meier curves.** Kaplan-Meier curves estimating the overall survival (OS) function for European GIA individuals in the validation dataset (n=28,545). Individuals were stratified into quartiles using each PLENC coordinate (e.g., "p25" denotes the bottom quartile) and OS curves were constructed using the standard Kaplan-Meier estimator with bootstrapping. The center lines are the means and the error bands are boostrapped 95% confidence intervals. See Supplementary Table 9 for the corresponding hazard ratios per standard deviation.

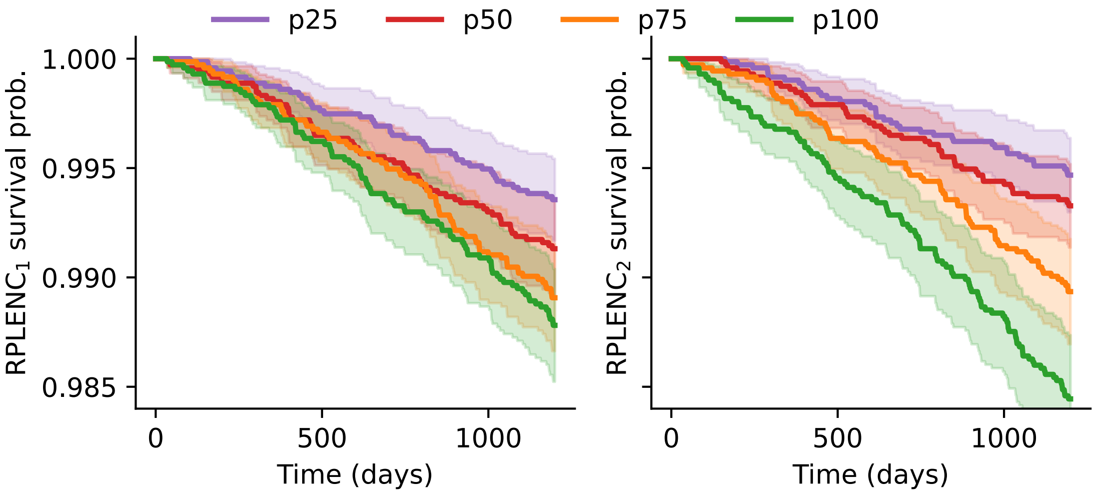

Supplementary Fig. 3: **RPLENCs Kaplan-Meier curves**. Kaplan-Meier curves estimating the overall survival (OS) function for European GIA individuals in the validation dataset (n=28,545). Individuals were stratified into quartiles using each RPLENC coordinate (e.g., "p25" denotes the bottom quartile) and OS curves were constructed using the standard Kaplan-Meier estimator with bootstrapping. The center lines are the means and the error bands are boostrapped 95% confidence intervals. See Supplementary Table 9 for the corresponding hazard ratios per standard deviation.

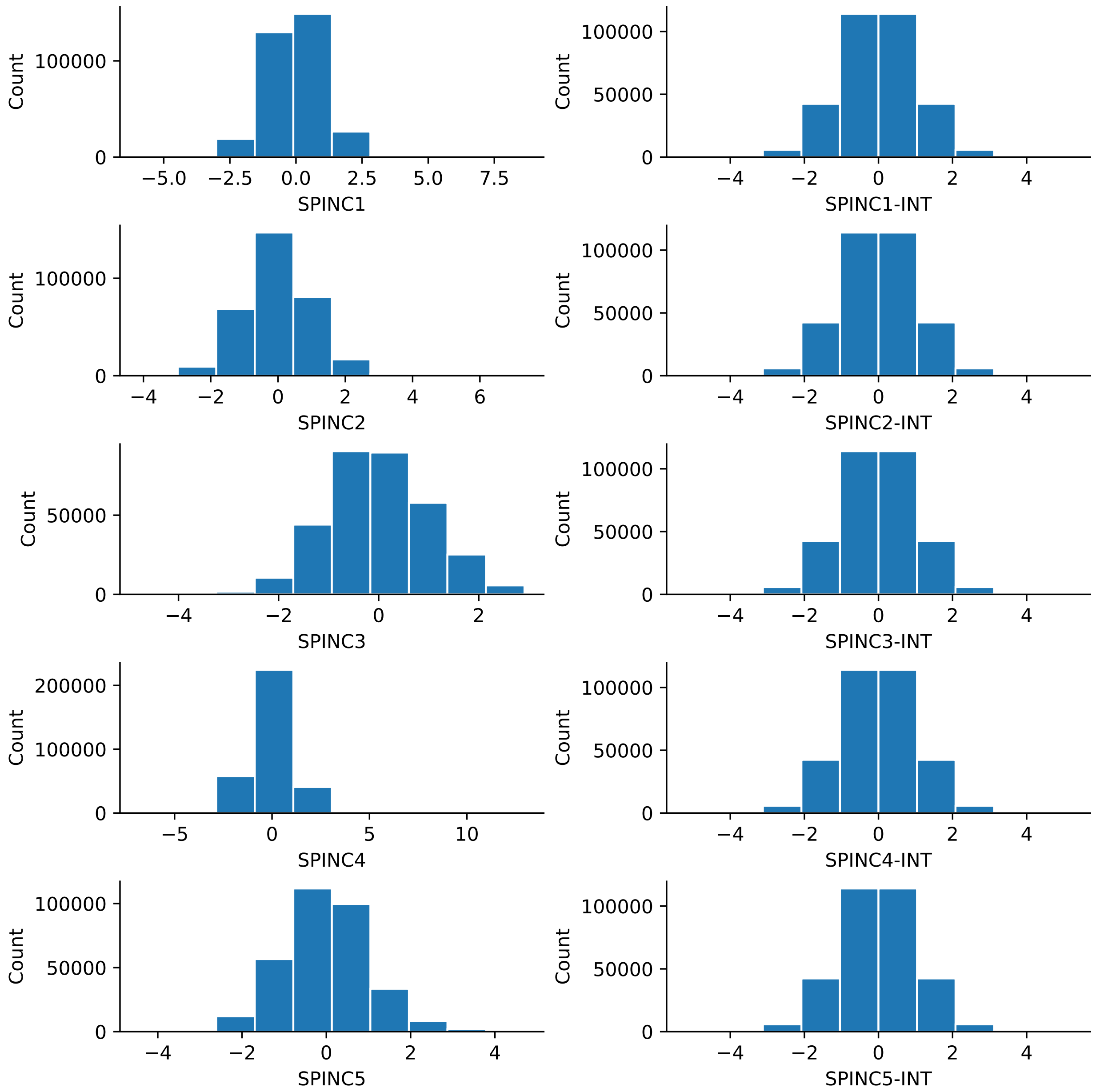

Supplementary Fig. 4: **Distribution of SPINCs and inverse-normal transformed SPINCs coordinates of UK Biobank individuals.** Histograms of 5 SPINCs coordinates and their inverse-normal transformations (INT).

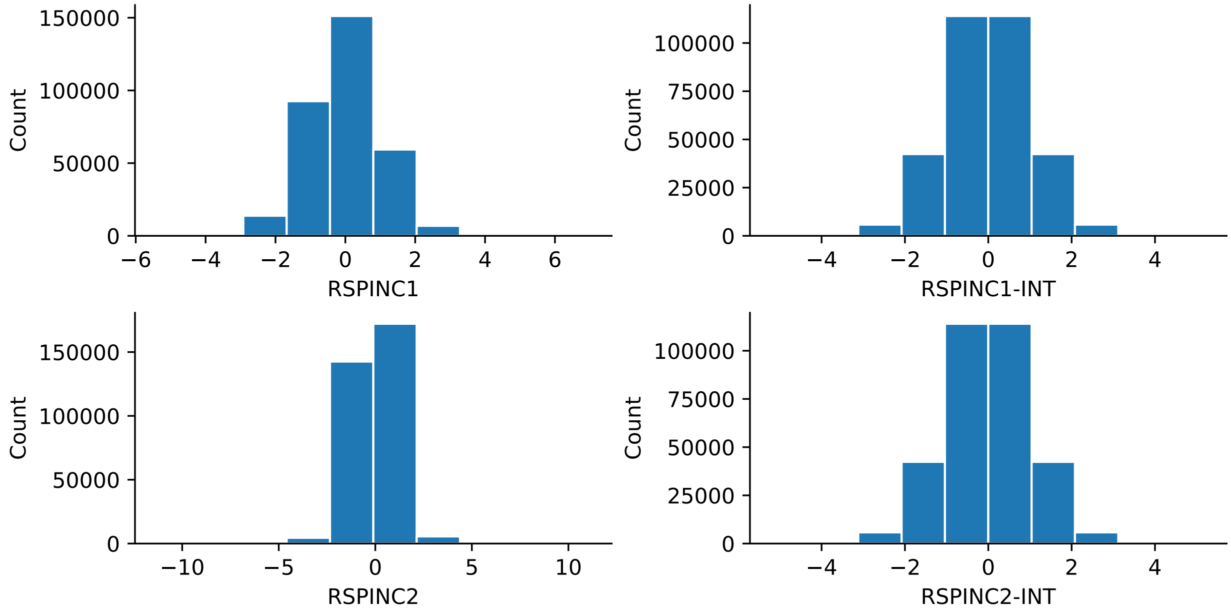

Supplementary Fig. 5: **Distribution of RSPINCs and inverse-normal transformed RSPINCs coordinates of UK Biobank individuals.** Histograms of 2 RSPINCs coordinates and their inverse-normal transformations (INT).

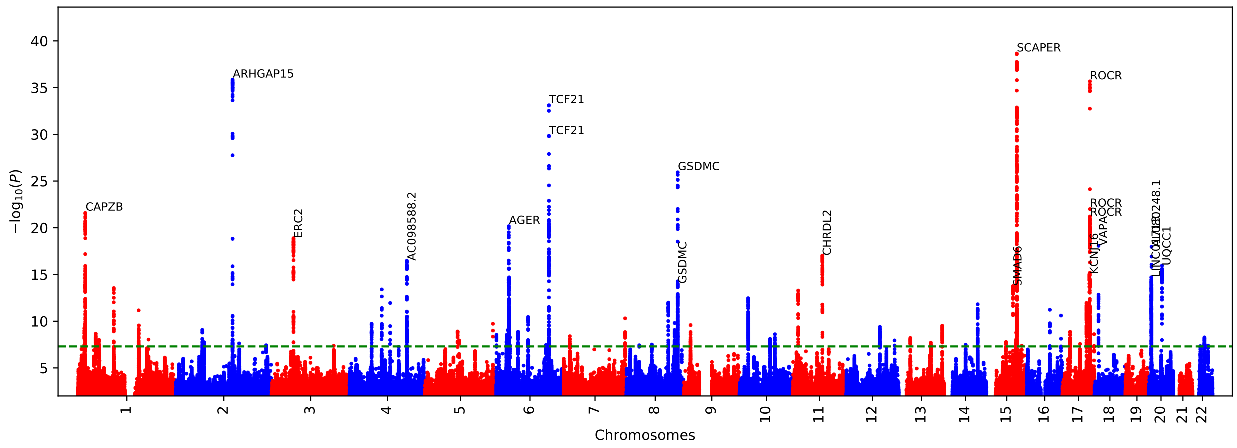

Supplementary Fig. 6: **$SPINC_1$ GWAS Manhattan plot.**

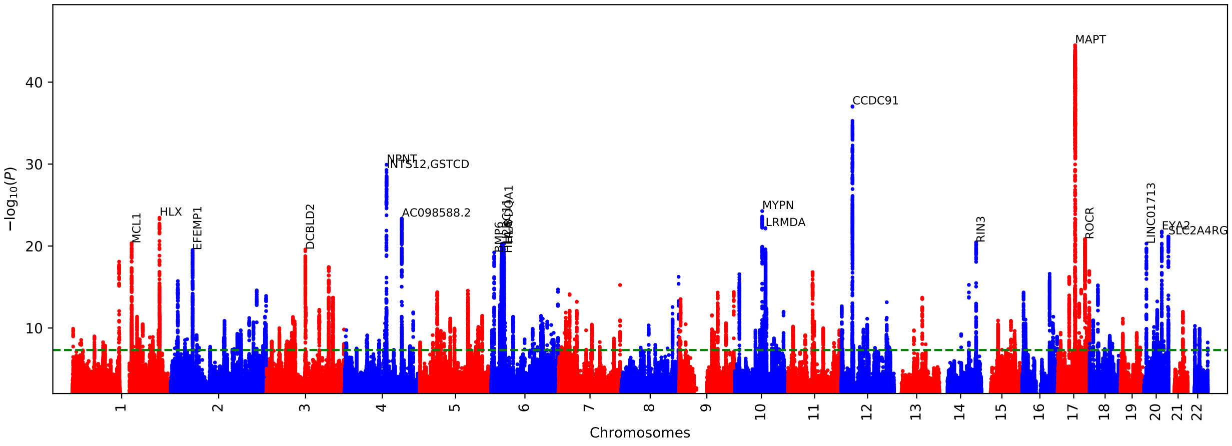

Supplementary Fig. 7: **SPINC$_2$ GWAS Manhattan plot.**

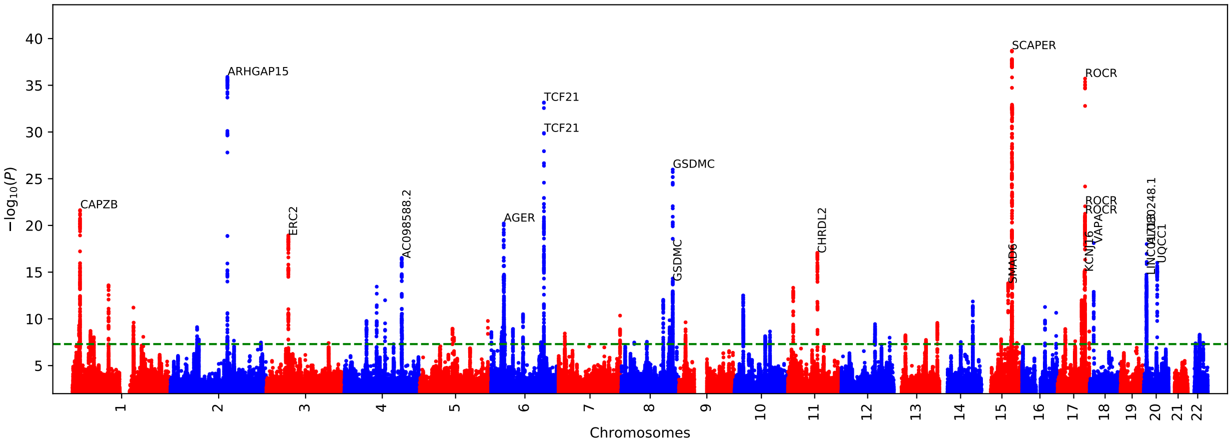

Supplementary Fig. 8: **SPINC$_3$ GWAS Manhattan plot.**

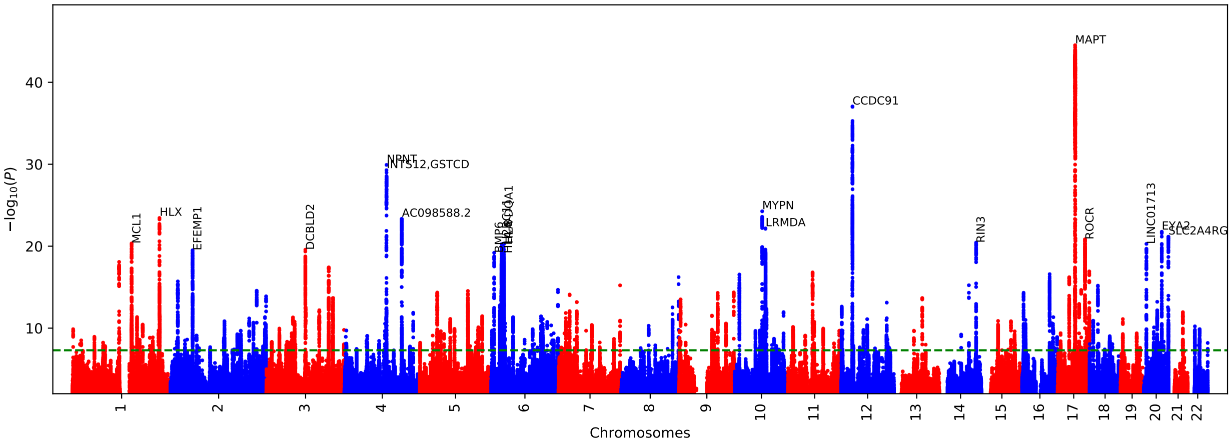

Supplementary Fig. 9: **SPINC$_4$ GWAS Manhattan plot.**

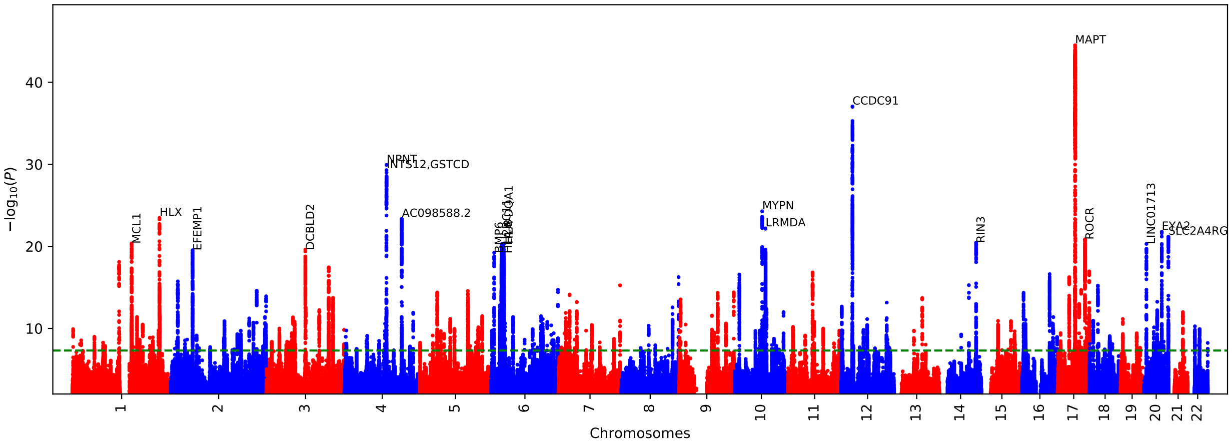

Supplementary Fig. 10: **$SPINC_5$ GWAS Manhattan plot.**

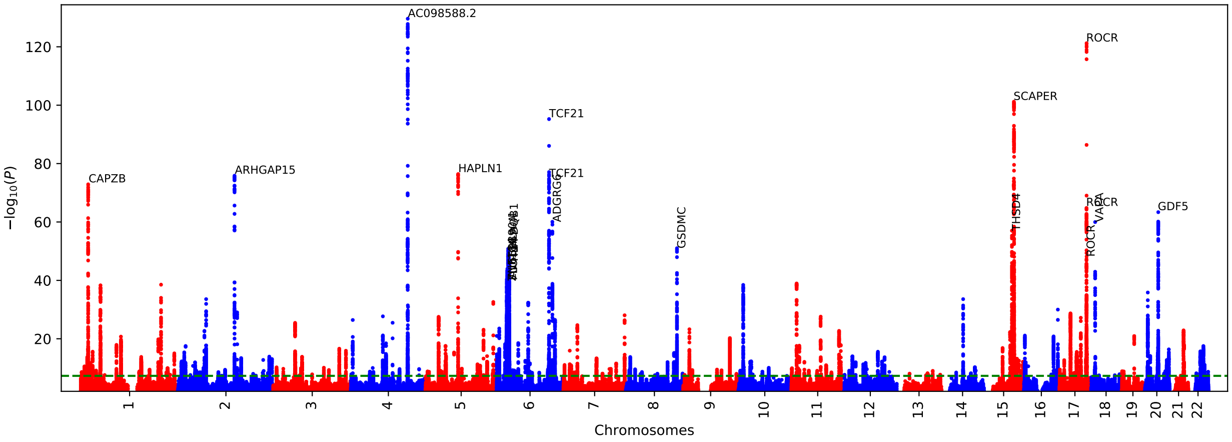

Supplementary Fig. 11: **$RSPINC_1$ GWAS Manhattan plot.**

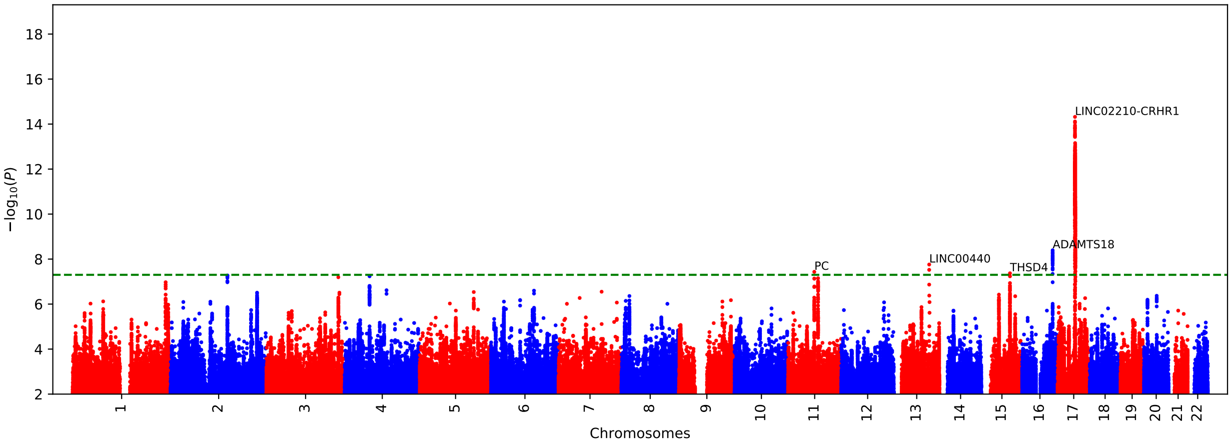

Supplementary Fig. 12: **$RSPINC_2$ GWAS Manhattan plot.**

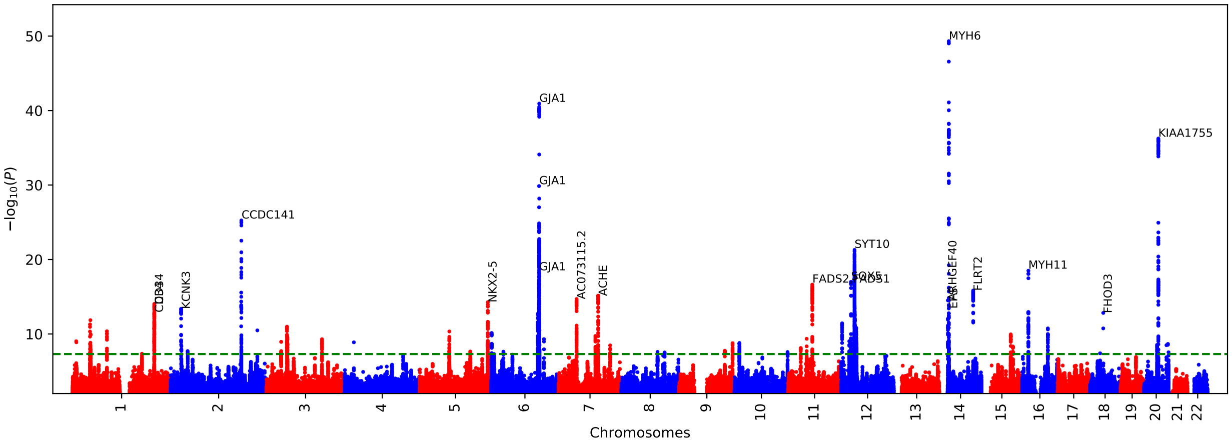

Supplementary Fig. 13: **PLENC$_1$ GWAS Manhattan plot.**

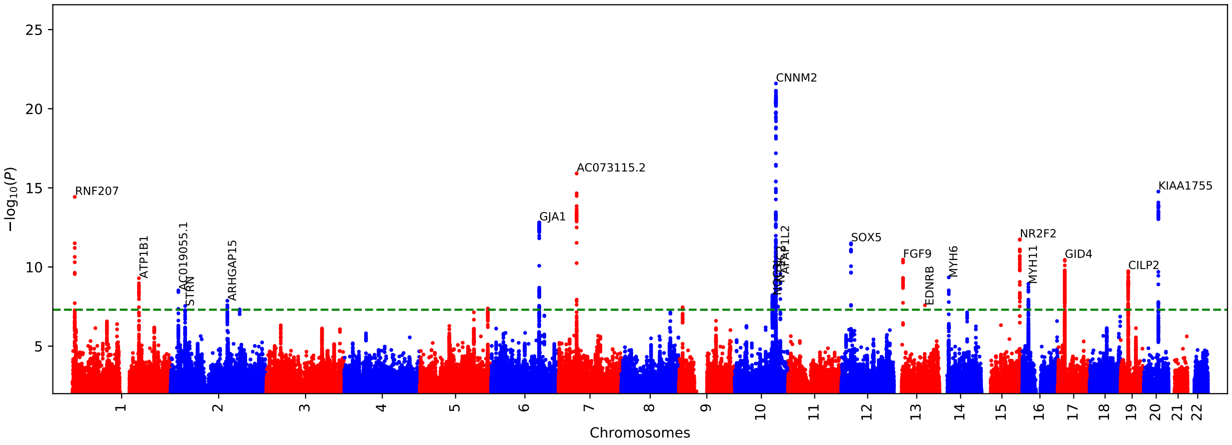

Supplementary Fig. 14: **PLENC$_2$ GWAS Manhattan plot.**

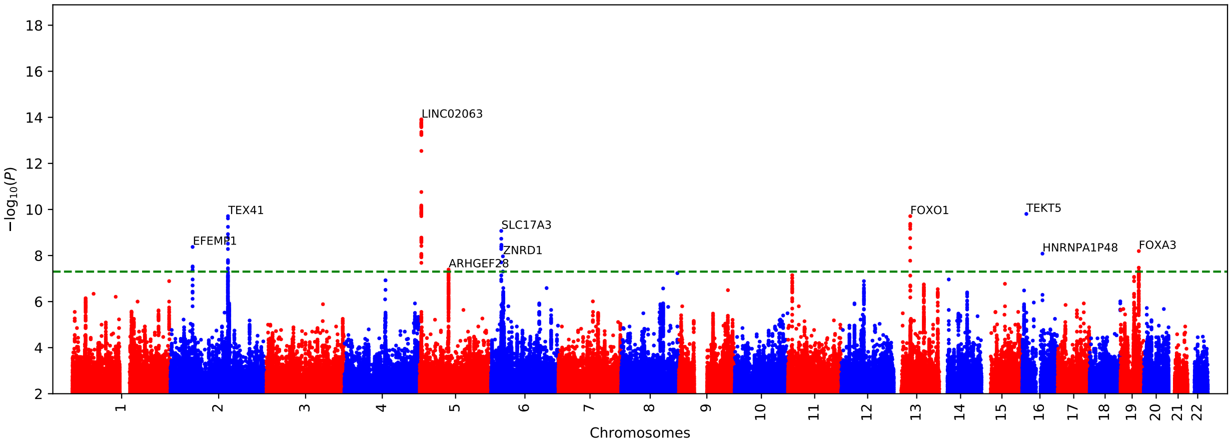

Supplementary Fig. 15: **PLENC$_3$ GWAS Manhattan plot.**

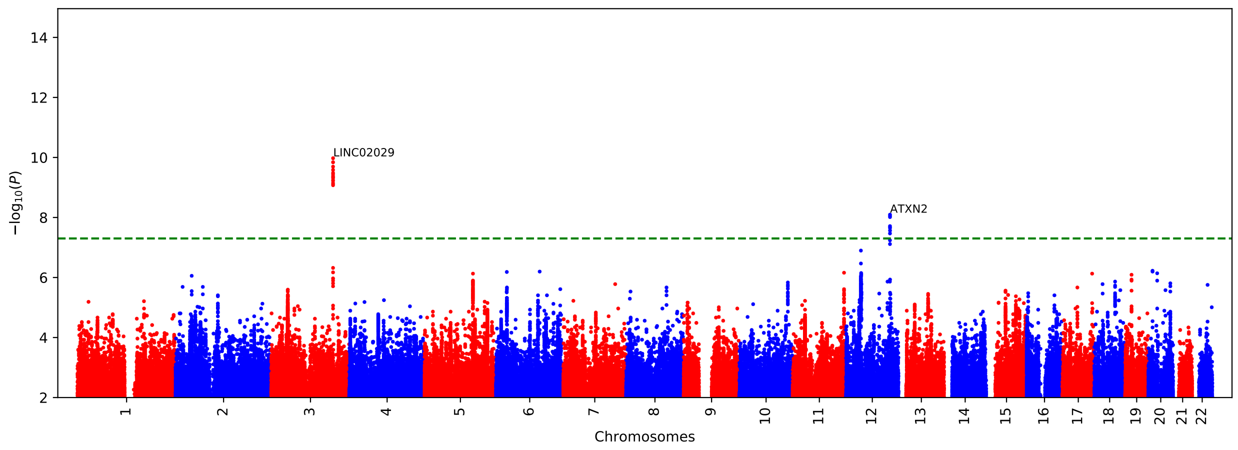

Supplementary Fig. 16: **PLENC$_4$ GWAS Manhattan plot.**

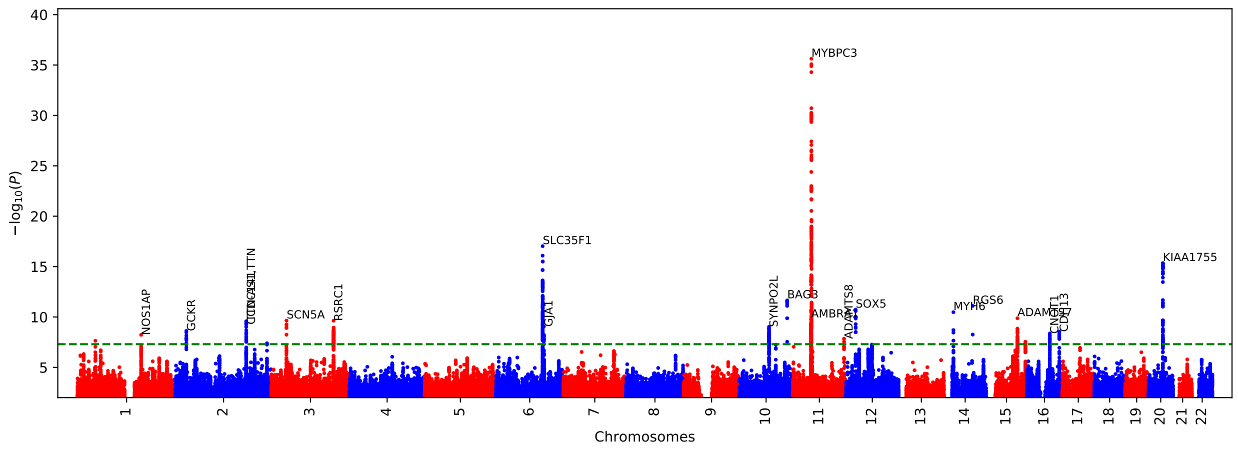

Supplementary Fig. 17: **PLENC$_5$ GWAS Manhattan plot.**

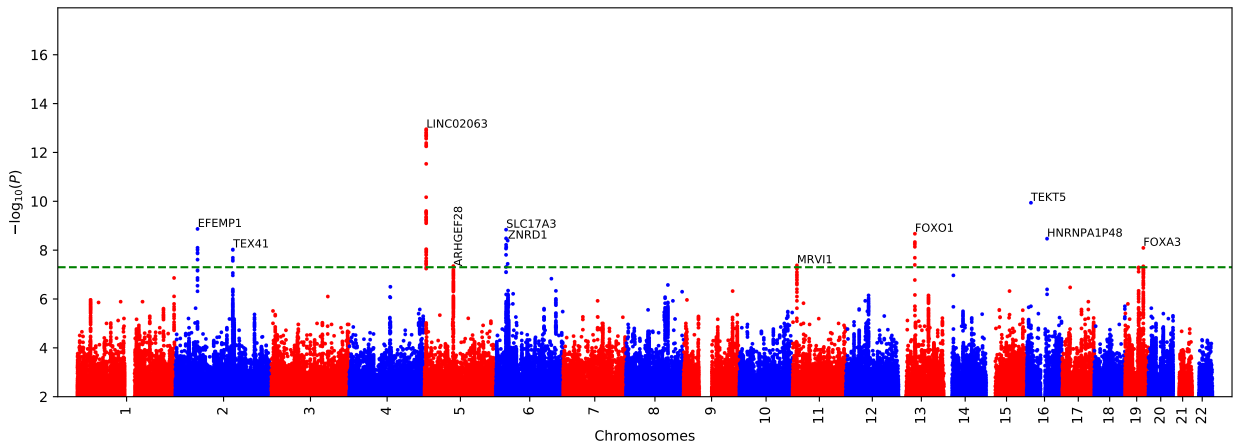

Supplementary Fig. 18: **RPLENC$_1$ GWAS Manhattan plot.**

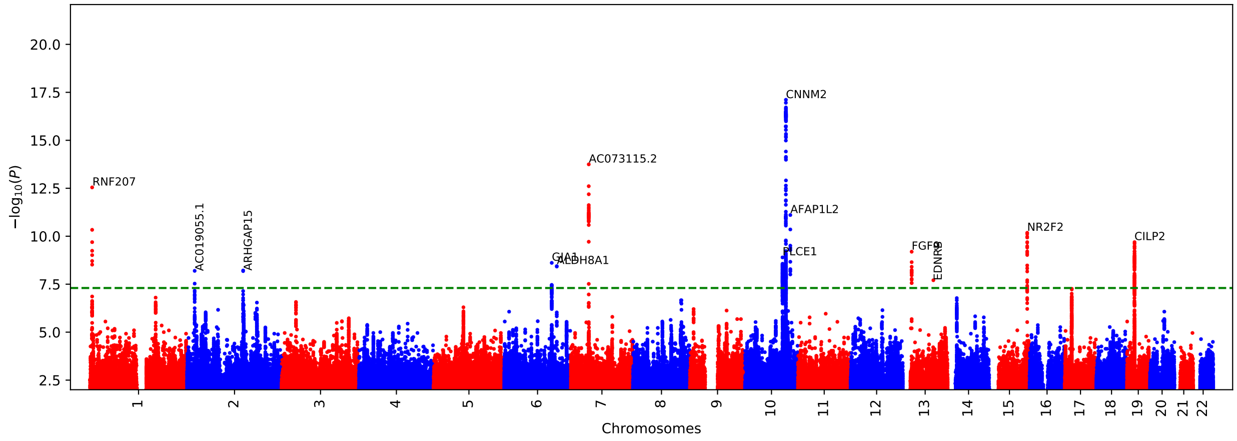

Supplementary Fig. 19: **RPLENC$_2$ GWAS Manhattan plot.**

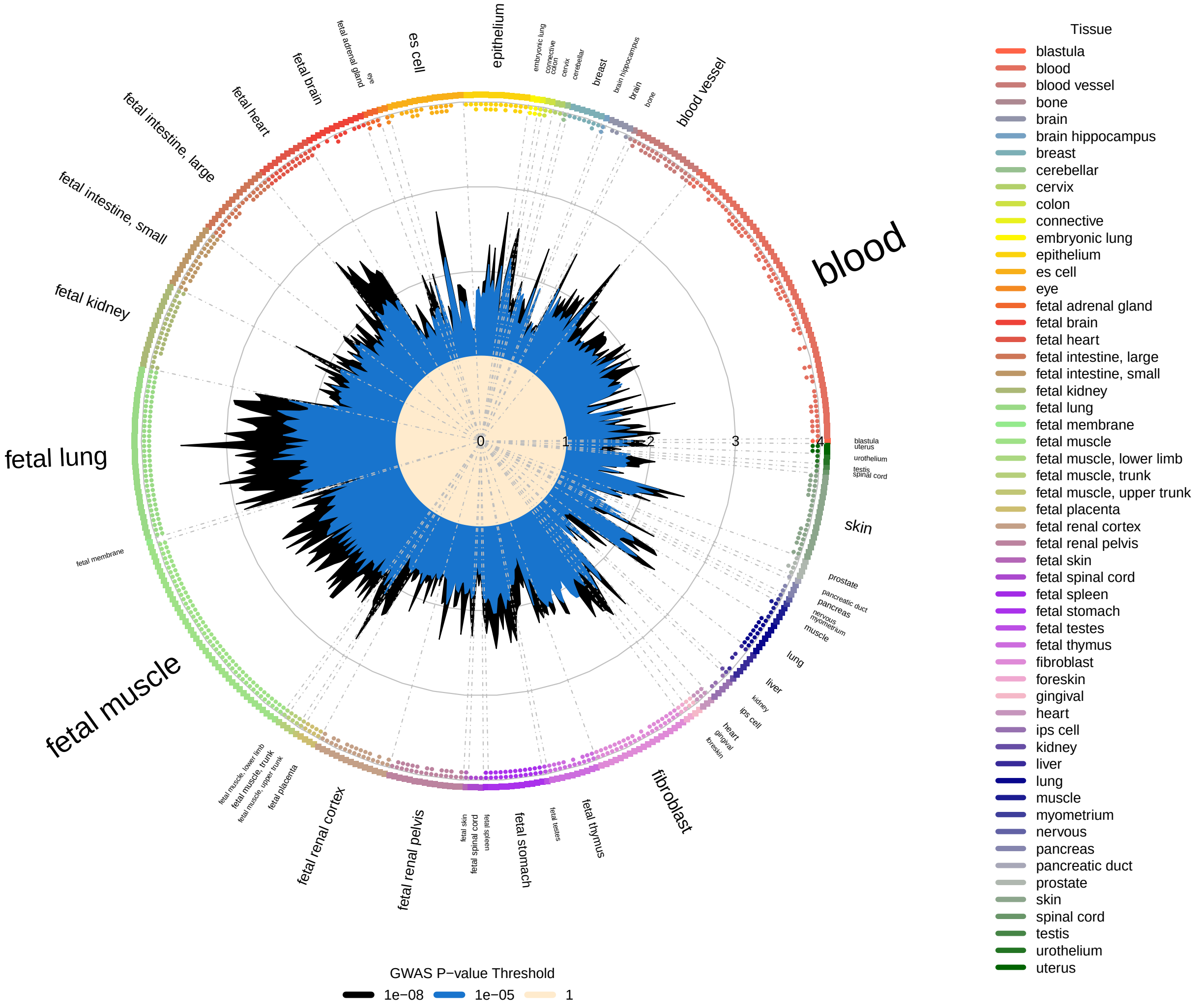

Supplementary Fig. 20: **Enrichment overlap of SPINC$_1$ GWAS with DNase I hotspots computed using GARFIELD.** Radial plot illustrates the enrichment (OR) in each cell type for different GWAS p-value thresholds ($P < 10^{-8}$ and 10^{-5}). In addition, the small dots on the outer side of the plot indicates enrichment significant level computed by GARFIELD for different significant level of 10^{-5}, 10^{-6}, 10^{-7}, and 10^{-8} in direction of outside to insider of plot (raw two-sided P-values are reported). GWAS p-values were obtained from BOLT-LMM using a two-sided test.

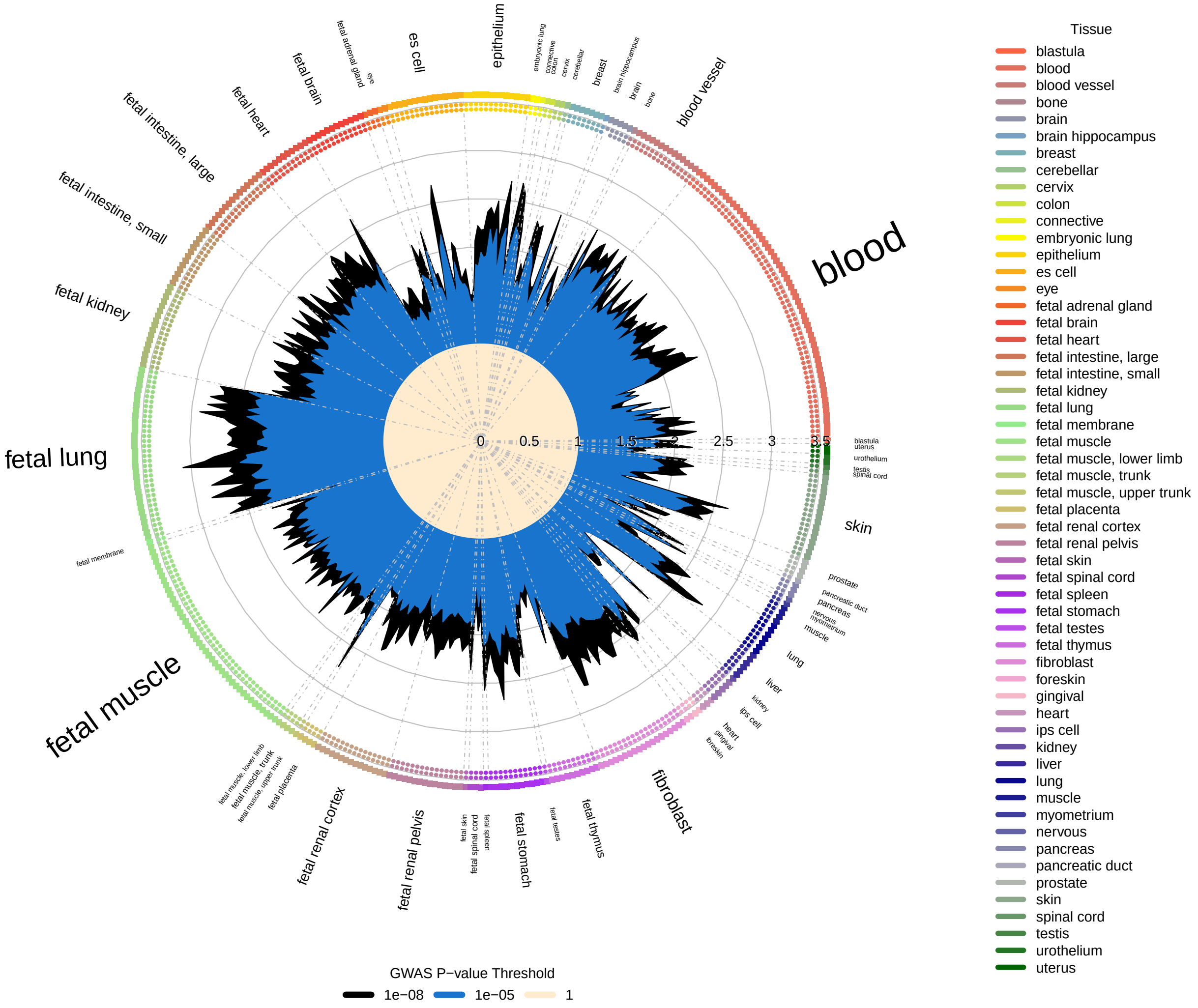

Supplementary Fig. 21: **Enrichment overlap of SPINC$_2$ GWAS with DNase I hotspots computed using GARFIELD.** Radial plot illustrates the enrichment (OR) in each cell type for different GWAS p-value thresholds ($P < 10^{-8}$ and 10^{-5}). In addition, the small dots on the outer side of the plot indicates enrichment significant level computed by GARFIELD for different significant level of 10^{-5}, 10^{-6}, 10^{-7}, and 10^{-8} in direction of outside to insider of plot (raw two-sided P-values are reported). GWAS p-values were obtained from BOLT-LMM using a two-sided test.

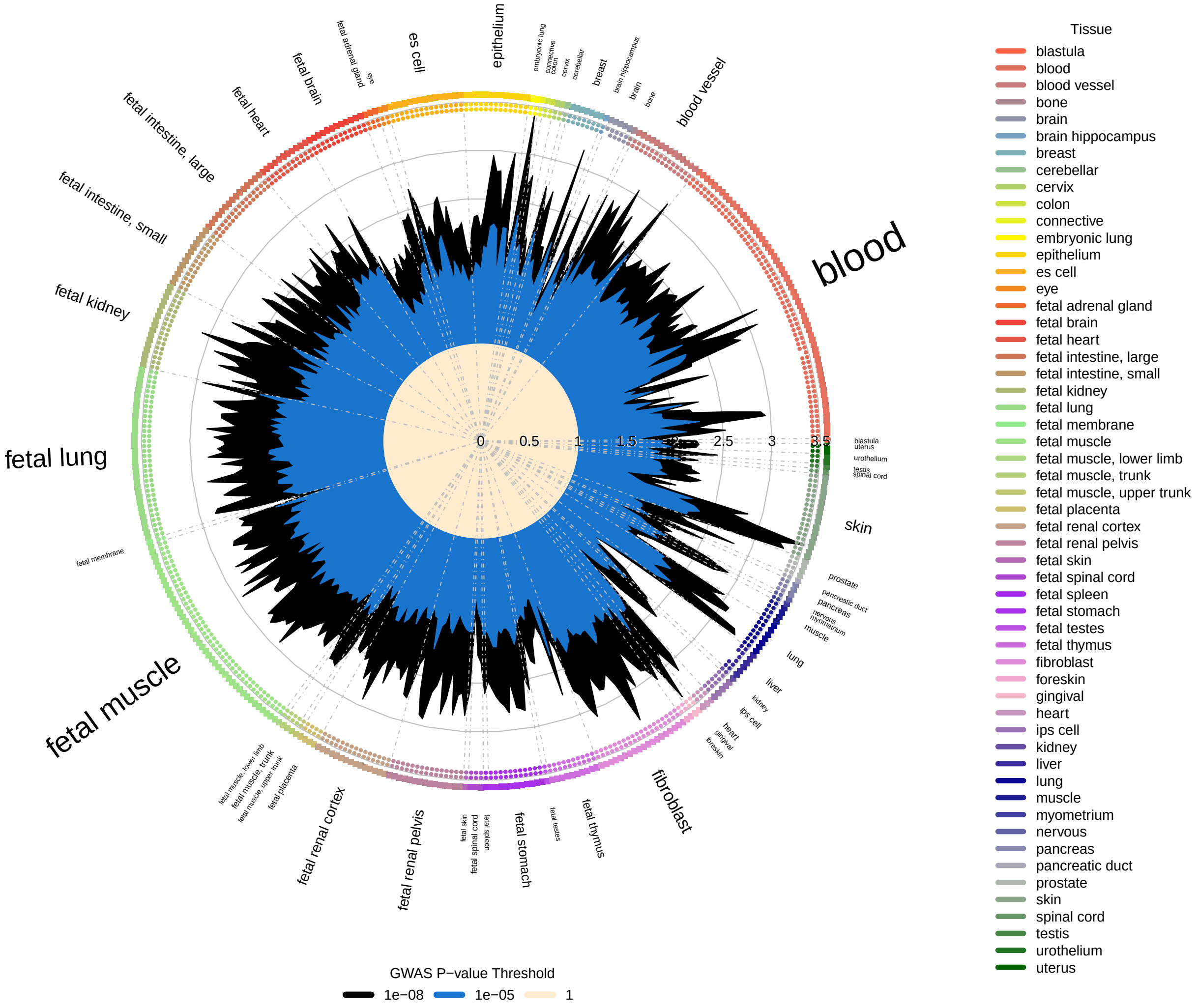

Supplementary Fig. 22: **Enrichment overlap of $SPINC_3$ GWAS with DNase I hotspots computed using GARFIELD.** Radial plot illustrates the enrichment (OR) in each cell type for different GWAS p-value thresholds ($P < 10^{-8}$ and 10^{-5}). In addition, the small dots on the outer side of the plot indicates enrichment significant level computed by GARFIELD for different significant level of 10^{-5}, 10^{-6}, 10^{-7}, and 10^{-8} in direction of outside to insider of plot (raw two-sided P-values are reported). GWAS p-values were obtained from BOLT-LMM using a two-sided test.

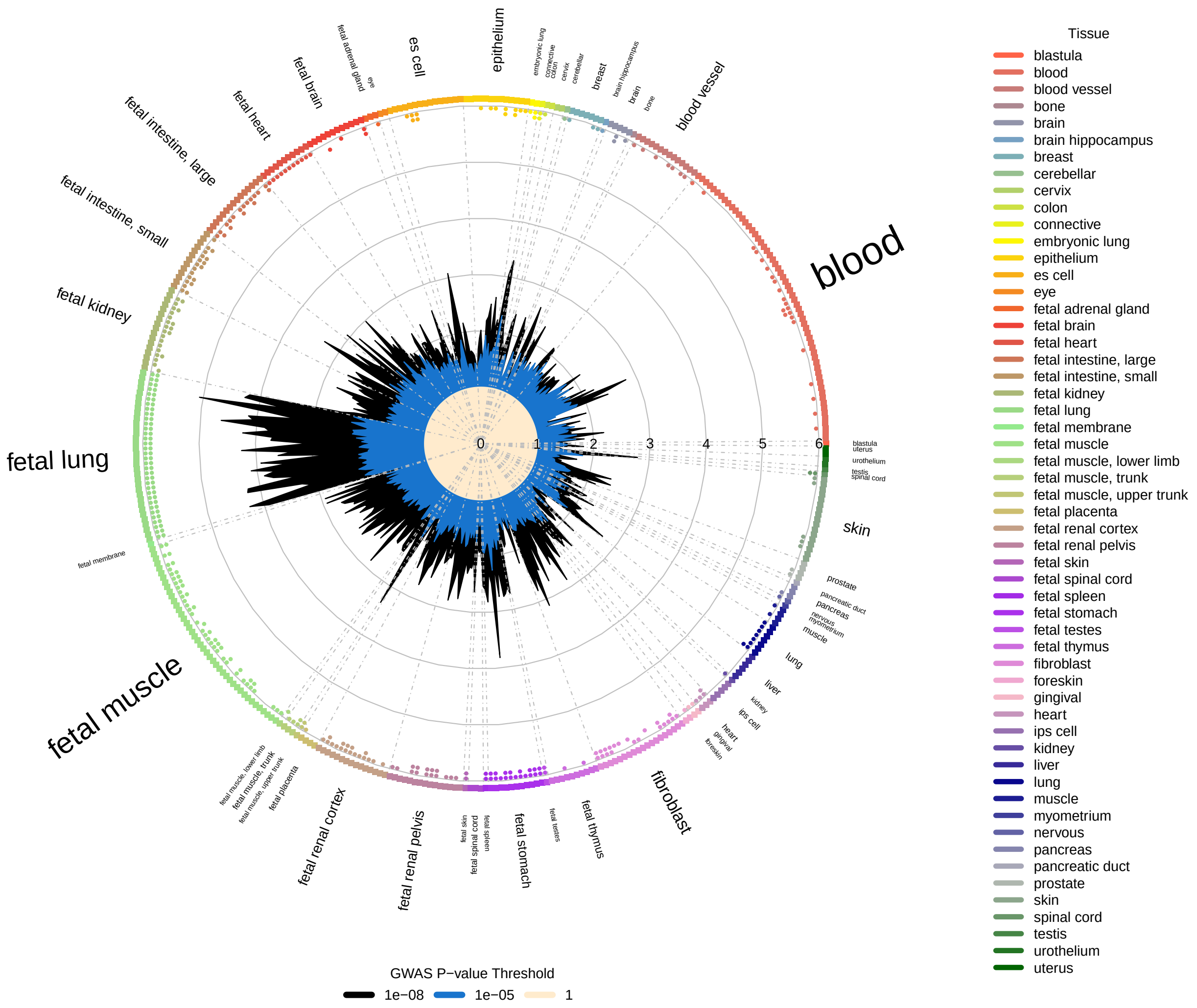

Supplementary Fig. 23: **Enrichment overlap of SPINC$_4$ GWAS with DNase I hotspots computed using GARFIELD.** Radial plot illustrates the enrichment (OR) in each cell type for different GWAS p-value thresholds ($P < 10^{-8}$ and 10^{-5}). In addition, the small dots on the outer side of the plot indicates enrichment significant level computed by GARFIELD for different significant level of 10^{-5}, 10^{-6}, 10^{-7}, and 10^{-8} in direction of outside to insider of plot (raw two-sided P-values are reported). GWAS p-values were obtained from BOLT-LMM using a two-sided test.

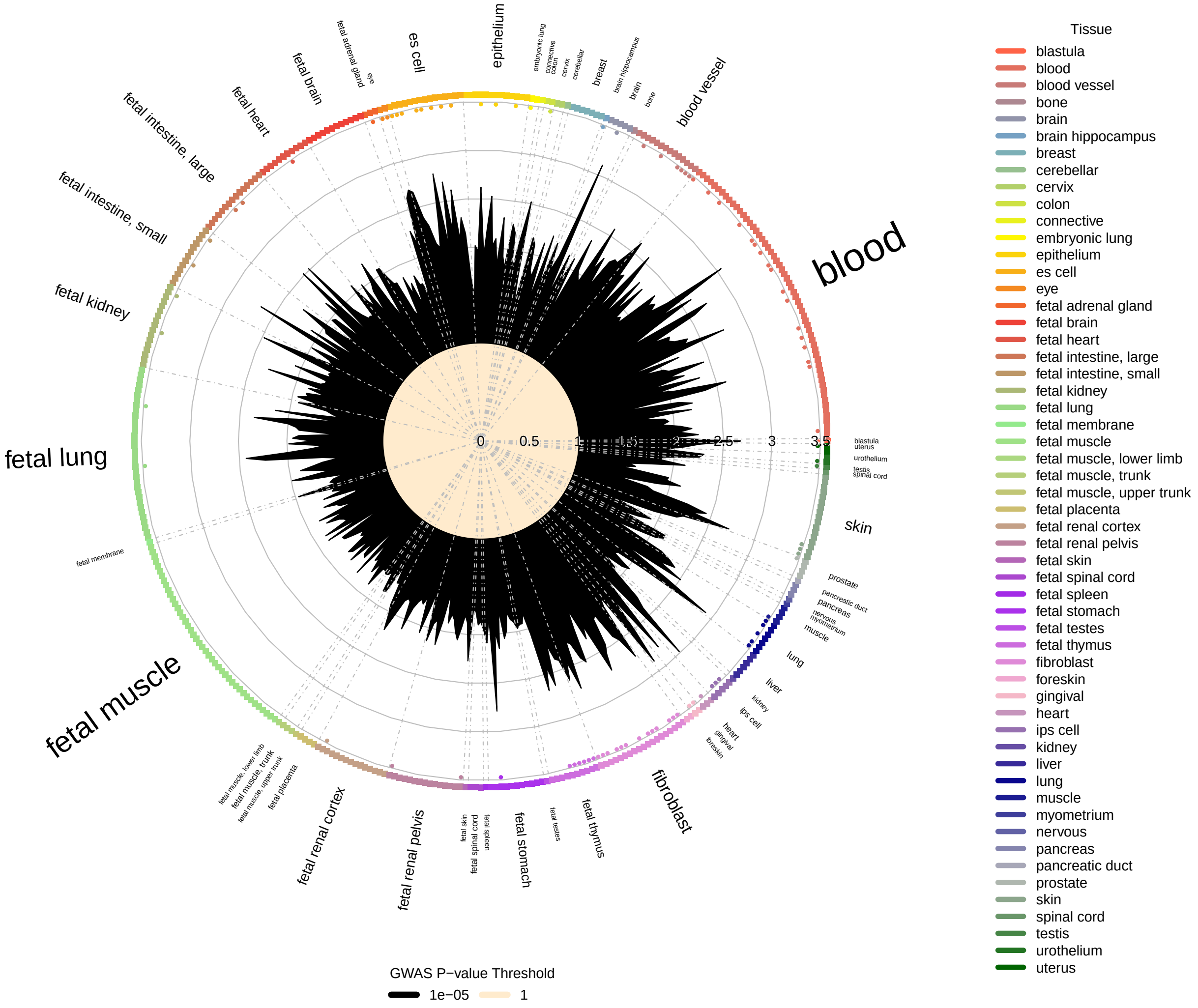

Supplementary Fig. 24: **Enrichment overlap of SPINC$_5$ GWAS with DNase I hotspots computed using GARFIELD.** Radial plot illustrates the enrichment (OR) in each cell type for different GWAS p-value thresholds ($P < 10^{-8}$ and 10^{-5}). In addition, the small dots on the outer side of the plot indicates enrichment significant level computed by GARFIELD for different significant level of 10^{-5}, 10^{-6}, 10^{-7}, and 10^{-8} in direction of outside to insider of plot (raw two-sided P-values are reported). GWAS p-values were obtained from BOLT-LMM using a two-sided test.

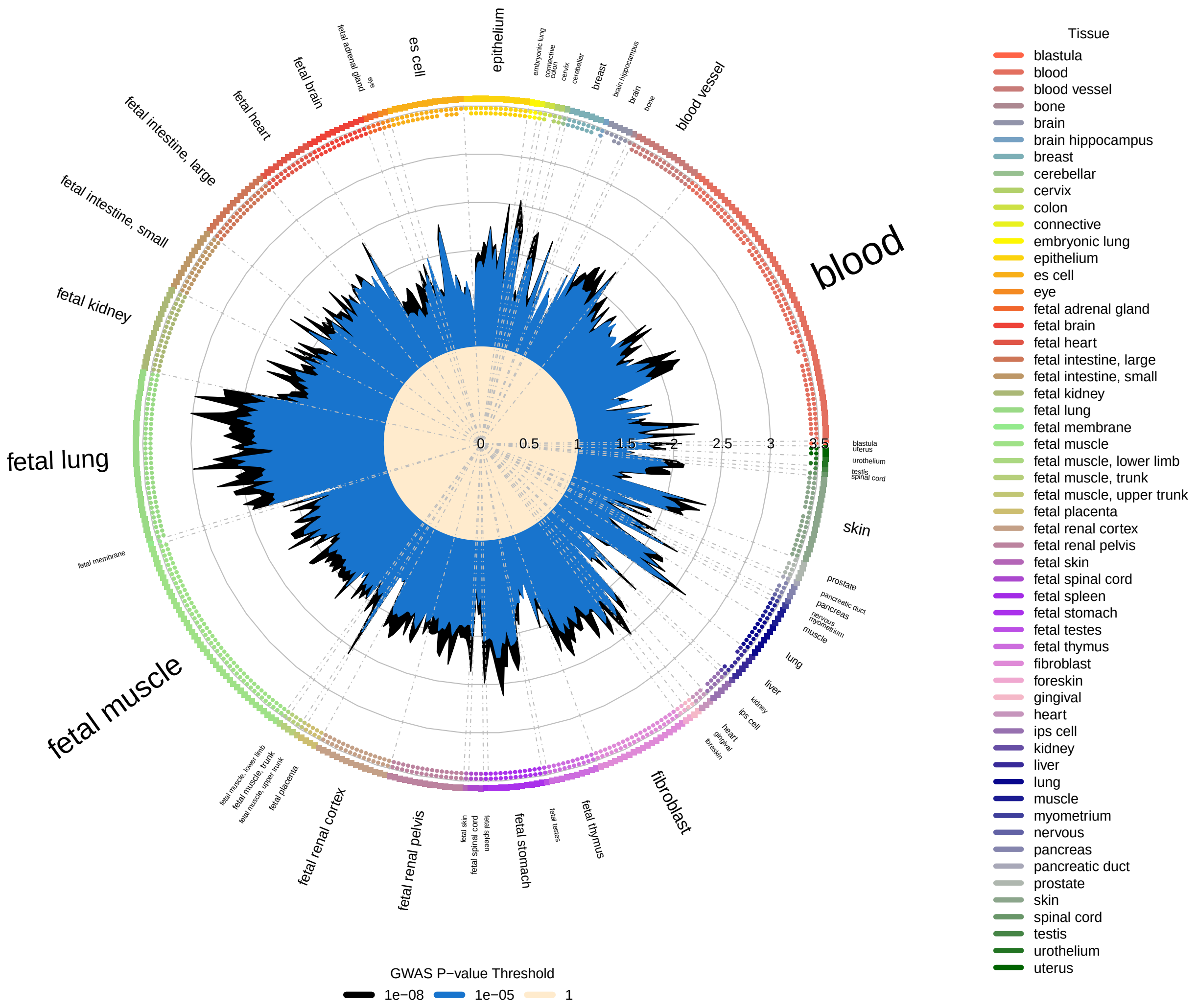

Supplementary Fig. 25: **Enrichment overlap of RSPINC$_1$ GWAS with DNase I hotspots computed using GARFIELD.** Radial plot illustrates the enrichment (OR) in each cell type for different GWAS p-value thresholds ($P < 10^{-8}$ and 10^{-5}). In addition, the small dots on the outer side of the plot indicates enrichment significant level computed by GARFIELD for different significant level of 10^{-5}, 10^{-6}, 10^{-7}, and 10^{-8} in direction of outside to insider of plot (raw two-sided P-values are reported). GWAS p-values were obtained from BOLT-LMM using a two-sided test.

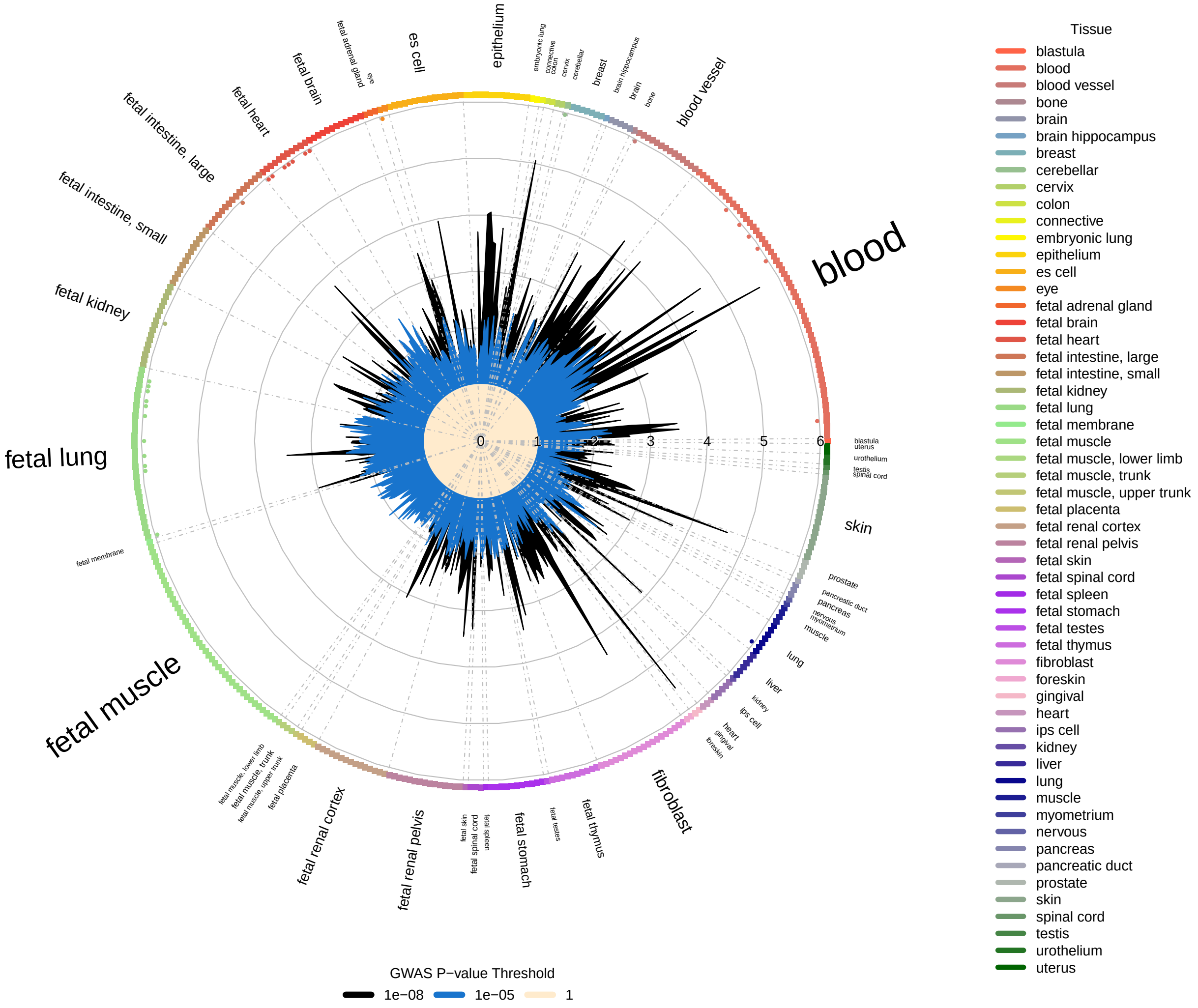

Supplementary Fig. 26: **Enrichment overlap of RSPINC$_2$ GWAS with DNase I hotspots computed using GARFIELD.** Radial plot illustrates the enrichment (OR) in each cell type for different GWAS p-value thresholds ($P < 10^{-8}$ and 10^{-5}). In addition, the small dots on the outer side of the plot indicates enrichment significant level computed by GARFIELD for different significant level of 10^{-5}, 10^{-6}, 10^{-7}, and 10^{-8} in direction of outside to insider of plot (raw two-sided P-values are reported). GWAS p-values were obtained from BOLT-LMM using a two-sided test.

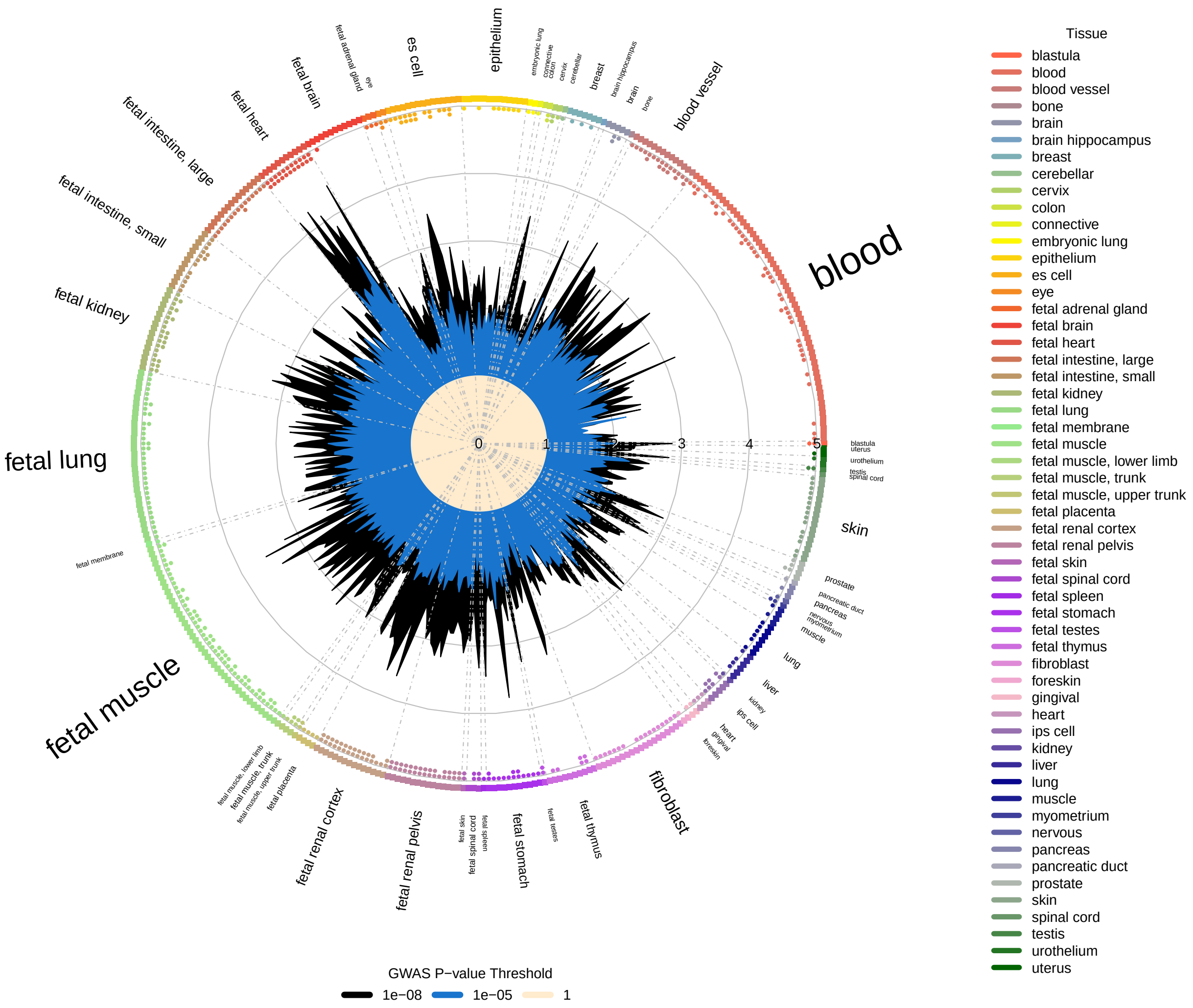

Supplementary Fig. 27: **Enrichment overlap of PLENC$_1$ GWAS with DNase I hotspots computed using GARFIELD.** Radial plot illustrates the enrichment (OR) in each cell type for different GWAS p-value thresholds (P $< 10^{-8}$ and 10^{-5}). In addition, the small dots on the outer side of the plot indicates enrichment significant level computed by GARFIELD for different significant level of 10^{-5}, 10^{-6}, 10^{-7}, and 10^{-8} in direction of outside to insider of plot (raw two-sided P-values are reported). GWAS p-values were obtained from BOLT-LMM using a two-sided test.

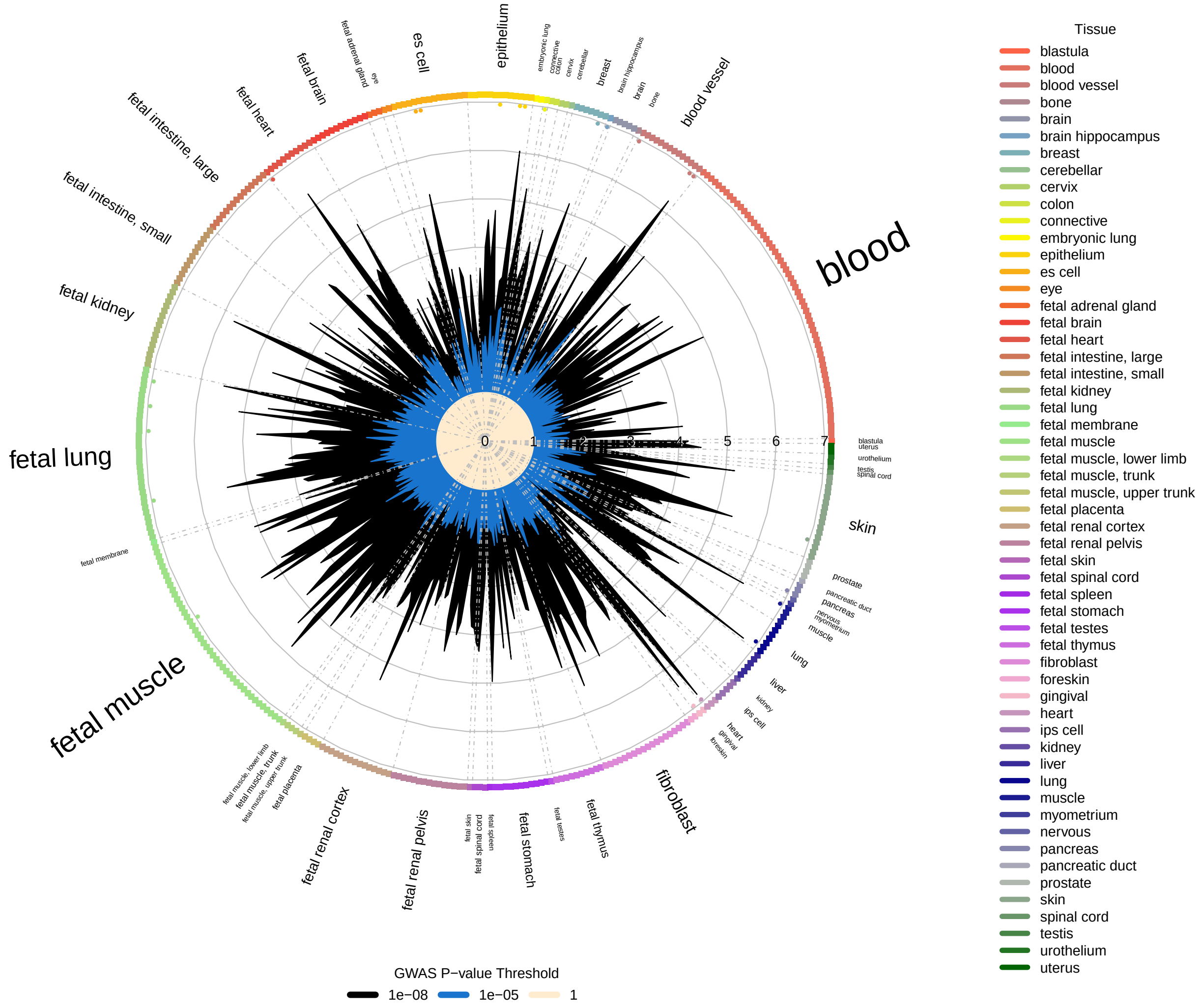

Supplementary Fig. 28: **Enrichment overlap of PLENC$_2$ GWAS with DNase I hotspots computed using GARFIELD.** Radial plot illustrates the enrichment (OR) in each cell type for different GWAS p-value thresholds ($P < 10^{-8}$ and 10^{-5}). In addition, the small dots on the outer side of the plot indicates enrichment significant level computed by GARFIELD for different significant level of 10^{-5}, 10^{-6}, 10^{-7}, and 10^{-8} in direction of outside to insider of plot (raw two-sided P-values are reported). GWAS p-values were obtained from BOLT-LMM using a two-sided test.

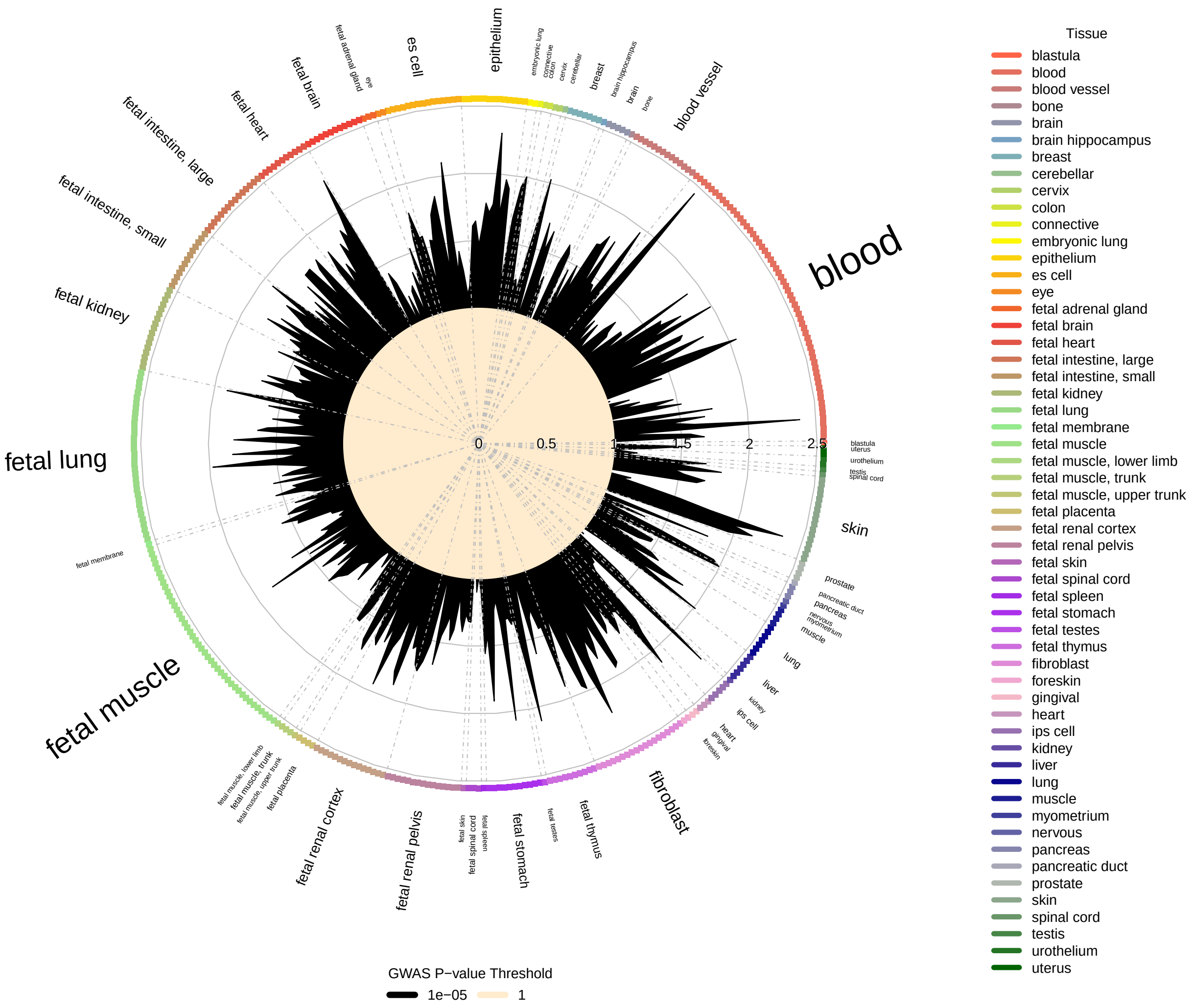

Supplementary Fig. 29: **Enrichment overlap of PLENC$_3$ GWAS with DNase I hotspots computed using GARFIELD.** Radial plot illustrates the enrichment (OR) in each cell type for different GWAS p-value thresholds (P $< 10^{-8}$ and 10^{-5}). In addition, the small dots on the outer side of the plot indicates enrichment significant level computed by GARFIELD for different significant level of 10^{-5}, 10^{-6}, 10^{-7}, and 10^{-8} in direction of outside to insider of plot (raw two-sided P-values are reported). GWAS p-values were obtained from BOLT-LMM using a two-sided test.

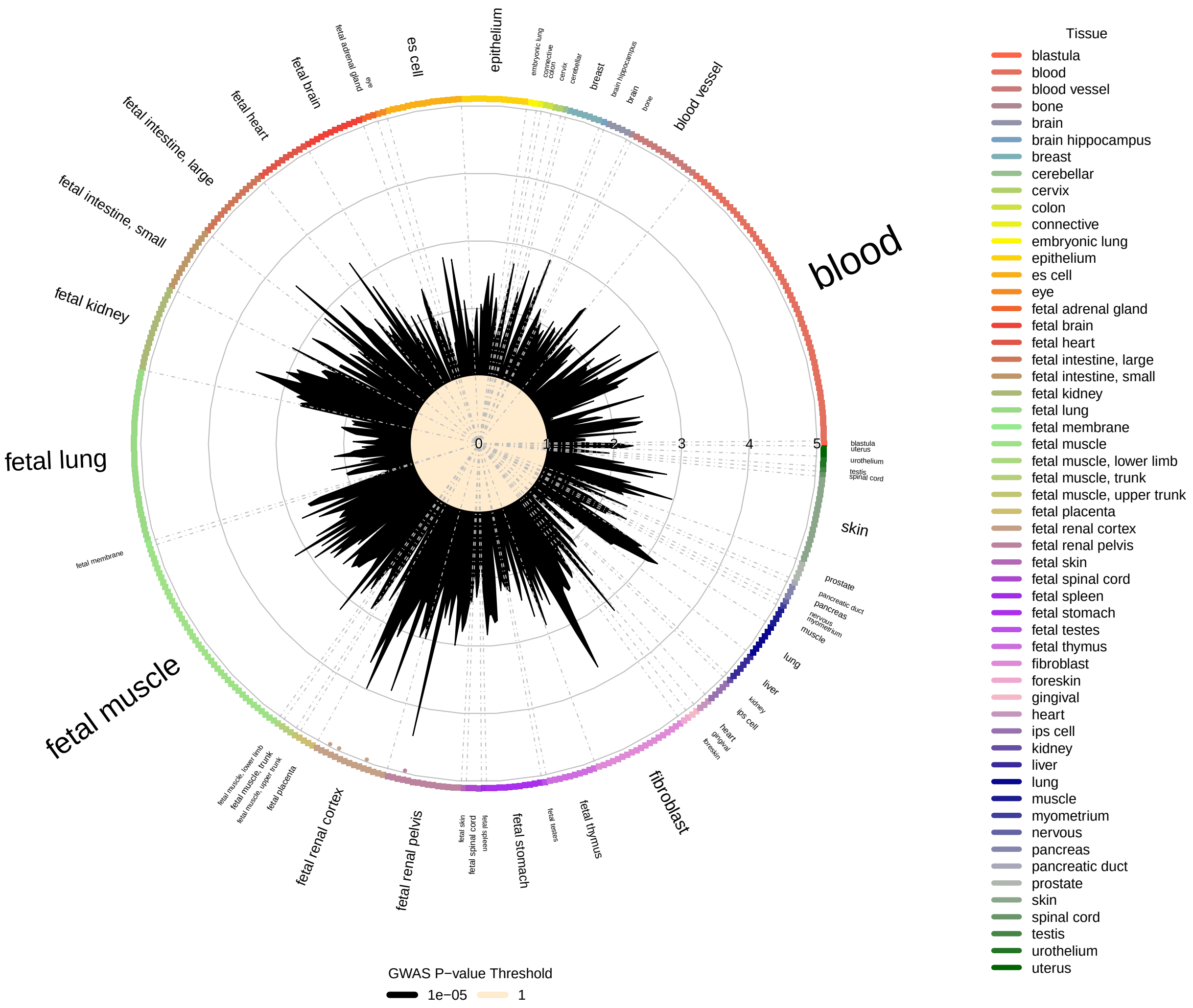

Supplementary Fig. 30: **Enrichment overlap of PLENC$_4$ GWAS with DNase I hotspots computed using GARFIELD.** Radial plot illustrates the enrichment (OR) in each cell type for different GWAS p-value thresholds ($P < 10^{-8}$ and 10^{-5}). In addition, the small dots on the outer side of the plot indicates enrichment significant level computed by GARFIELD for different significant level of 10^{-5}, 10^{-6}, 10^{-7}, and 10^{-8} in direction of outside to insider of plot (raw two-sided P-values are reported). GWAS p-values were obtained from BOLT-LMM using a two-sided test.

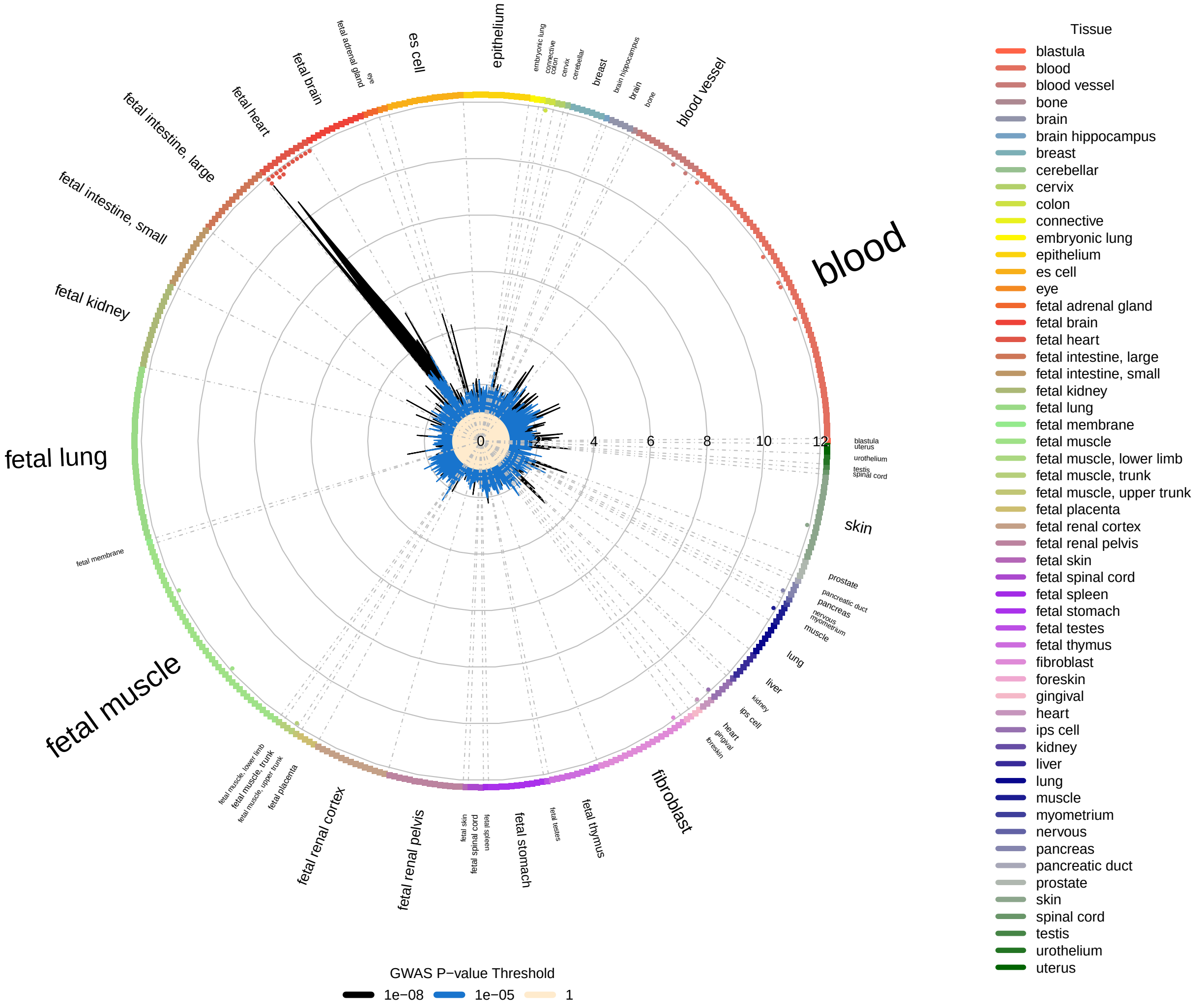

Supplementary Fig. 31: **Enrichment overlap of PLENC$_5$ GWAS with DNase I hotspots computed using GARFIELD.** Radial plot illustrates the enrichment (OR) in each cell type for different GWAS p-value thresholds ($P < 10^{-8}$ and 10^{-5}). In addition, the small dots on the outer side of the plot indicates enrichment significant level computed by GARFIELD for different significant level of 10^{-5}, 10^{-6}, 10^{-7}, and 10^{-8} in direction of outside to insider of plot (raw two-sided P-values are reported). GWAS p-values were obtained from BOLT-LMM using a two-sided test.

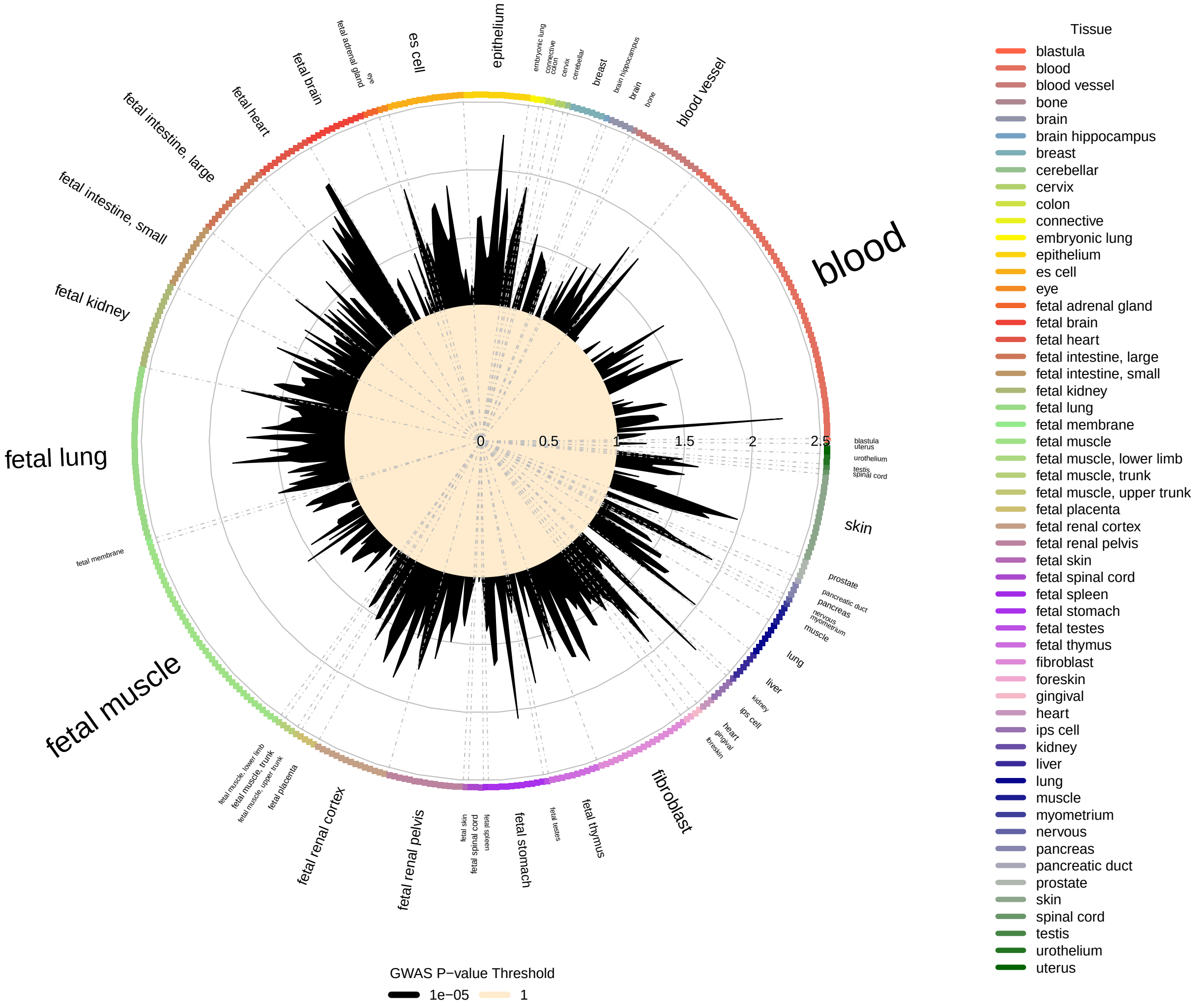

Supplementary Fig. 32: **Enrichment overlap of RPLENC$_1$ GWAS with DNase I hotspots computed using GARFIELD.** Radial plot illustrates the enrichment (OR) in each cell type for different GWAS p-value thresholds (P $< 10^{-8}$ and 10^{-5}). In addition, the small dots on the outer side of the plot indicates enrichment significant level computed by GARFIELD for different significant level of 10^{-5}, 10^{-6}, 10^{-7}, and 10^{-8} in direction of outside to insider of plot (raw two-sided P-values are reported). GWAS p-values were obtained from BOLT-LMM using a two-sided test.

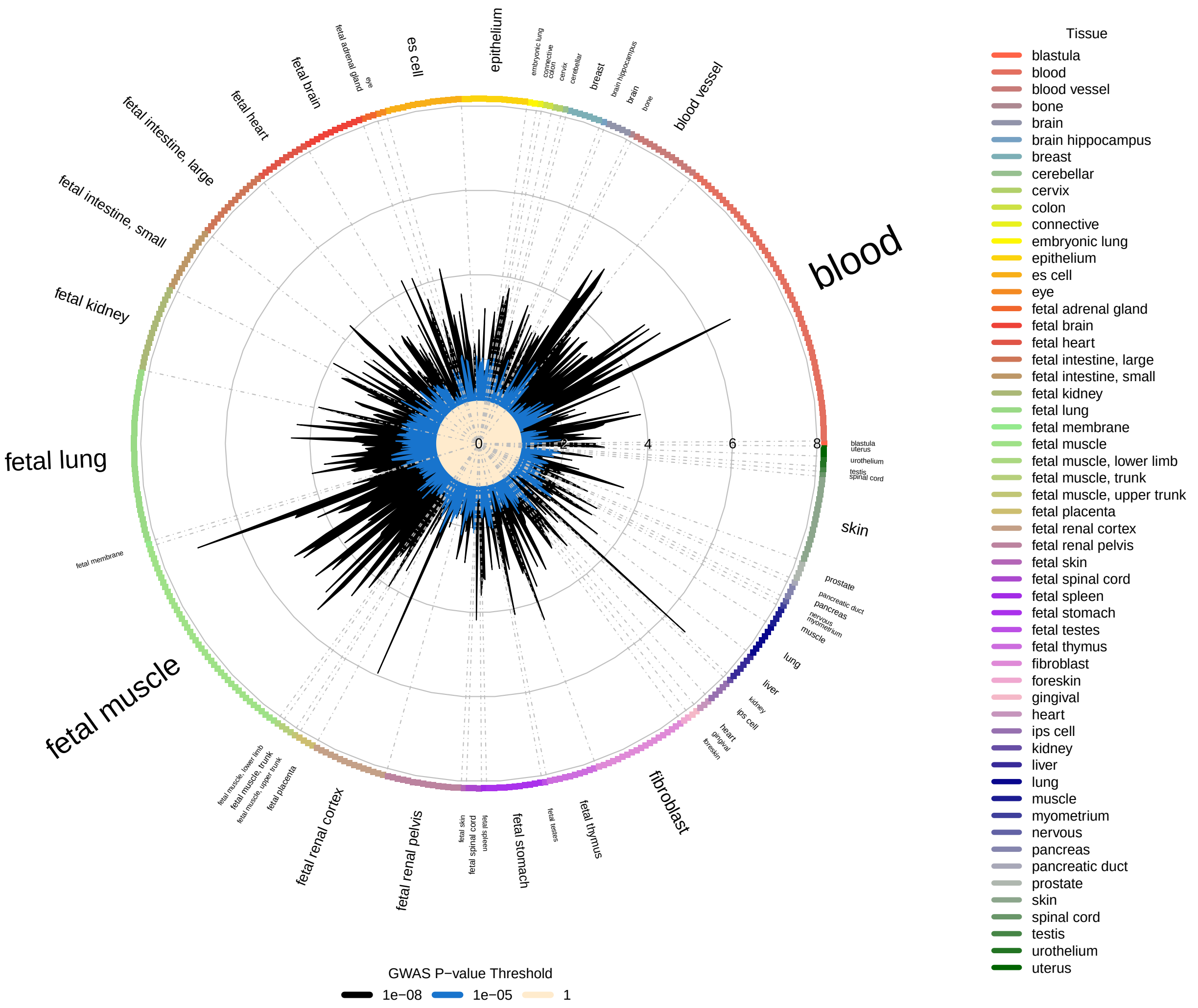

Supplementary Fig. 33: **Enrichment overlap of RPLENC_2 GWAS with DNase I hotspots computed using GARFIELD.** Radial plot illustrates the enrichment (OR) in each cell type for different GWAS p-value thresholds ($P < 10^{-8}$ and 10^{-5}). In addition, the small dots on the outer side of the plot indicates enrichment significant level computed by GARFIELD for different significant level of 10^{-5}, 10^{-6}, 10^{-7}, and 10^{-8} in direction of outside to insider of plot (raw two-sided P-values are reported). GWAS p-values were obtained from BOLT-LMM using a two-sided test.

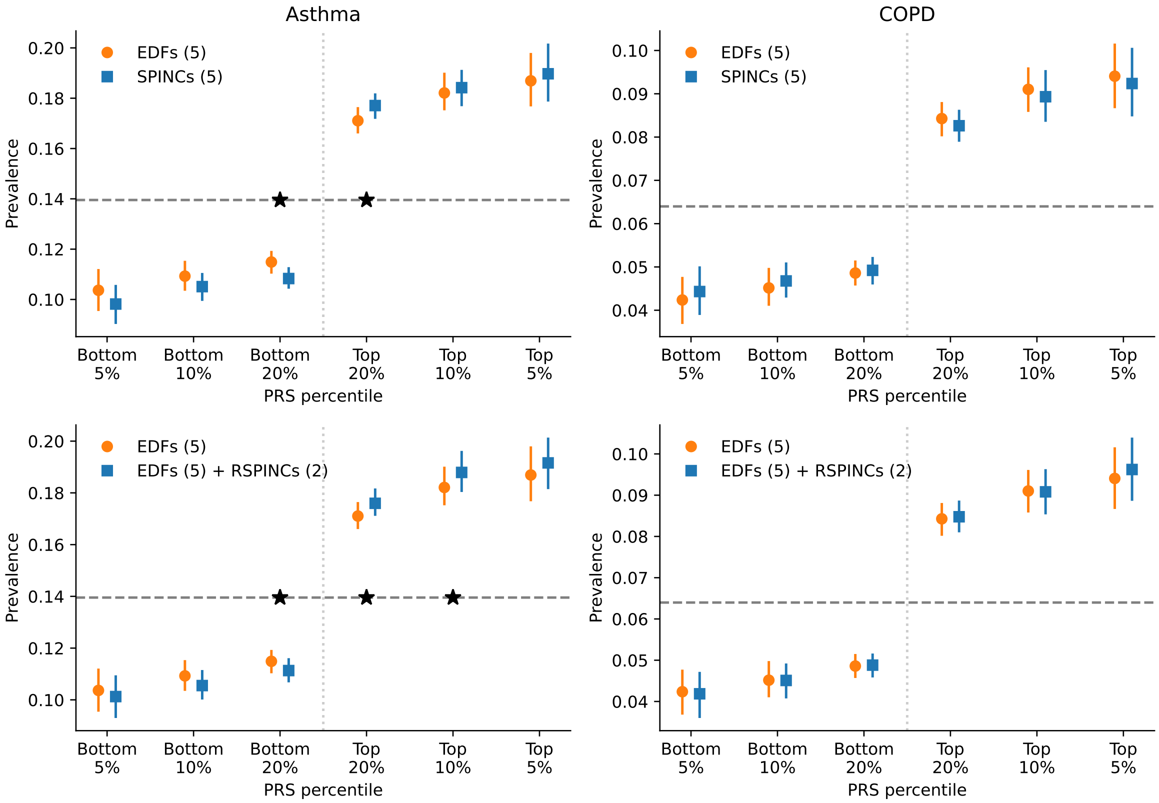

Supplementary Fig. 34: **PRS using SPINCs and RSPINCs in UK Biobank with inverse normal transformation.** Combined PRS for asthma and COPD using three sets of intermediate PRS, five EDFs, five SPINCs, and five EDFs + two RSPINCs, after applying inverse-normal transformation on all. Each set of PRS is combined by a linear model trained using the target phenotype labels and the prevalence of the phenotypes in the top and bottom 5%, 10%, and 20% PRS individuals is evaluated in a separate evaluation set. In all figures, solid vertical intervals represent 95% confidence intervals generated by statistical bootstrapping (300 repetitions), and the center points are the bootstrapping means. The horizontal dashed lines show the total prevalence in the evaluation set. Star (*) symbols indicate a statistically significant difference between the two methods using *paired* bootstrapping (300 repetitions) with 95% confidence (i.e. two-sided $P < 0.05$). Lower is better for the bottom percentiles; higher is better for the top percentiles.

Supplementary Tables

Method	Optimizer	Learning rate	Batch size
SPINCs	Adam	1E-4	16
RSPINCs	Adam	1E-4	16
PLENCs	Adam	1E-4	16
RPLENCs	Adam	1E-3	32

Supplementary Table 1: **Overview of the final hyperparameters used for each method.** Hyperparameter search was run over the learning rates of {1E-5, 1E-4, 1E-3} and the batch sizes of {16, 32, 64}. See also "SPINCs model architecture", "RSPINCs model architecture", "PLENCs model architecture", and "RPLENCs model architecture" in Supplementary Notes.

Latent Dimension	SPINCs	PCA	RSPINCs
0	-	-	0.105144
1	0.063391	0.102179	0.038280
2	0.015881	0.051901	0.027203
3	0.008463	0.027312	0.019421
4	0.006265	0.017987	0.014800
5	0.005251	0.012641	0.012816
6	0.005263	0.009538	0.011725

Supplementary Table 2: **Reconstruction error of spirograms based on MSE.**

Latent Dimension	PLENCs	PCA	RPLENCs
1	0.161438	0.304929	0.107090
2	0.085320	0.167717	0.054795
3	0.055498	0.091606	0.042532
4	0.044633	0.053183	0.041056
5	0.039191	0.037153	0.041439

Supplementary Table 3: **Reconstruction error of PPGs based on MSE.**

	age	sex	height	BMI	smoker
SPINC_1-residual	-0.00	-0.00	0.00	0.03	0.01
SPINC_2-residual	-0.17	-0.01	0.01	-0.02	-0.05
SPINC_3-residual	-0.08	-0.06	-0.02	-0.05	-0.02
SPINC_4-residual	0.11	-0.01	-0.01	-0.02	0.03
SPINC_5-residual	0.13	0.04	0.01	0.04	0.04
RSPINC_1-residual	0.18	-0.02	-0.02	-0.01	0.04
RSPINC_2-residual	0.06	0.06	0.04	0.05	0.03

Supplementary Table 4: **Pearson correlation of (R)SPINCs residuals with lung-function-related covariates.** After residualizing the EDFs from the (R)SPINCS, they retain some information about the covariates.

Fields group	P-value
Asthma (e.g. medical conditions self-report)	1.18E-133
Quality of spirograms collected (e.g. the number of spirometry measurements made)	5.75E-62
Breathing issues (e.g. wheeze or whistling in the chest in last year)	3.83E-54
Cognitive function (e.g. reaction time)	1.43E-22
Hayfever, allergic rhinitis or eczema (e.g. medical conditions self-report)	6.99E-20
Location (e.g. assessment center in Leeds)	1.22E-17

Supplementary Table 5: **Selected groups of fields significantly correlated with (R)SPINCs after residualizing EDFs and covariates.** We note that the high correlation with "location" could be due to technical issues in particular assessment centers or ascertainment bias. The two-sided P-values of Pearson correlations were computed using the exact distribution of Pearson R under the null hypothesis with independent normal distributions, as implemented in SciPy.

See the attached Excel table.

Supplementary Table 6: **Pearson correlation of (R)SPINCs residuals with UKB tabular fields.** After residualizing the EDFs and covariates from the (R)SPINCS, they still retain information about respiratory diseases such as asthma and allergic rhinitis, breathing issues, quality of spirograms, and cognitive function. The two-sided P-values of Pearson correlations were computed using the exact distribution of Pearson R under the null hypothesis with independent normal distributions, as implemented in SciPy.

Fields group	P-value
Pulse rate	<5.00e-300
Pulse rate, automated reading	5.65E-284
Systolic blood pressure, automated reading	1.17E-153
Pulse wave reflection index	1.12E-112
Pulse rate, automated reading	2.03E-77
Diastolic blood pressure, automated reading	6.74E-67
Position of the shoulder on the pulse waveform	3.98E-50
Position of the pulse wave peak	4.96E-42
ECG, heart rate	1.98E-35

Supplementary Table 7: **Selected groups of fields significantly correlated with (R)PLENCs after residualizing EDFs and covariates.** The two-sided P-values of Pearson correlations were computed using the exact distribution of Pearson R under the null hypothesis with independent normal distributions, as implemented in SciPy.

See the attached Excel table.

Supplementary Table 8: **Pearson correlation of (R)PLENCs residuals with UKB tabular fields.** After residualizing the EDFs and covariates from the PLENCs, they still retain information about cardiovascular system such as pulse rate, systolic and diastolic blood pressure, ECG heart rate. The two-sided P-values of Pearson correlations were computed using the exact distribution of Pearson R under the null hypothesis with independent normal distributions, as implemented in SciPy.

Risk	Hazard Ratio	Lower 95% CI	Upper 95% CI	P
$SPINC_1$	1.08565	1.0516	1.1208	4.16E-07
$SPINC_2$	1.13457	1.1010	1.1692	1.74E-16
$SPINC_3$	0.67943	0.6534	0.7065	1.57E-83
$SPINC_4$	1.07763	1.0449	1.1114	2.00E-06
$SPINC_5$	1.00962	0.9794	1.0408	5.37E-01
$RSPINC_1$	0.98222	0.9527	1.0127	2.49E-01
$RSPINC_2$	1.06364	1.0316	1.0967	7.81E-05
FEV_1	0.63971	0.6148	0.6656	8.10E-108
FVC	0.68493	0.6558	0.7153	2.15E-65
PEF	0.75200	0.7275	0.7773	7.72E-64
FEV_1/FVC	0.80562	0.7859	0.8258	1.07E-65
$FEF_{25-75\%}$	0.68917	0.6630	0.7164	2.23E-79
$PLENC_1$	0.87865	0.8339	0.9258	1.24E-06
$PLENC_2$	0.76070	0.7208	0.8029	2.83E-23
$PLENC_3$	1.07235	1.0177	1.1299	8.82E-03
$PLENC_4$	1.01382	0.9615	1.0690	0.611623
$PLENC_5$	0.99256	0.9419	1.0459	0.779784
$RPLENC_1$	1.05166	0.9974	1.1088	4.15E-20
$RPLENC_2$	1.27541	1.2109	1.3434	2.43E-03
Absence of Notch	1.07081	1.0245	1.1192	2.43E-03
Position of Notch	1.12913	1.0766	1.1842	5.89E-07
Position of Peak	1.11444	1.0536	1.1788	1.54E-04
Position of Shoulder	1.14546	1.0847	1.2096	1.04E-06
Peak to Peak Time	0.96973	0.9202	1.0219	2.50E-01

Supplementary Table 9: **Survival analysis hazard ratios per 1 standard deviation for SPINC, RSPINC, PLENC, RPLENC, and EDF risk scores.** Note that the $SPINC_1$ ($p = 0.0129$), $PLENC_1$ ($p = 0.0033$), $PLENC_2$ ($p = 0.0264$), and "Position of Notch" ($p = 0.0162$) models fail the proportional-hazards (PH) assumption and thus should be interpreted as hazard over time. The PH assumption holds for all other models. The P-values are from testing the null hypothesis (Hazard Ratio = 1) under the Cox PH model, as implemented in the "survival" R package.

Phenotype	S-LDSC Intercept	S-LDSC Attenuation Ratio	S-LDSC SNP-heritability
$SPINC_1$	1.0224 (0.0181)	0.0278 (0.0225)	0.1302 (0.0071)
$SPINC_2$	1.0466 (0.0294)	0.0294 (0.0186)	0.2481 (0.0132)
$SPINC_3$	1.0431 (0.0161)	0.0401 (0.0150)	0.1604 (0.0066)
$SPINC_4$	1.0289 (0.0147)	0.0591 (0.0300)	0.0746 (0.0055)
$SPINC_5$	1.0019 (0.0103)	0.0073 (0.0390)	0.0428 (0.0029)
Spiro PCA_1	1.0503 (0.0171)	0.0381 (0.0129)	0.1975 (0.0074)
Spiro PCA_2	1.0137 (0.0154)	0.0174 (0.0195)	0.1281 (0.0074)
Spiro PCA_3	1.0223 (0.0096)	0.1513 (0.0649)	0.0221 (0.003)
Spiro PCA_4	1.0223 (0.0113)	0.1577 (0.0803)	0.0194 (0.003)
Spiro PCA_5	1.0076 (0.0092)	0.0964 (0.1167)	0.0114 (0.0024)
FEV_1	1.0444 (0.0181)	0.0349 (0.0142)	0.1884 (0.0079)
FVC	1.0497 (0.0171)	0.0379 (0.0131)	0.1961 (0.0074)
PEF	1.0227 (0.015)	0.0291 (0.0192)	0.1222 (0.0063)
FEV_1/FVC	1.0511 (0.0242)	0.0395 (0.0187)	0.2026 (0.0101)
$FEF_{25_75\%}$	1.0733 (0.0285)	0.0448 (0.0175)	0.2445 (0.0115)
$RSPINC_1$	1.0231 (0.0237)	0.0219 (0.0225)	0.1615 (0.0113)
$RSPINC_2$	1.0113 (0.0114)	0.0404 (0.0408)	0.0446 (0.0034)
$PLENC_1$	1.0130 (0.0115)	0.0360 (0.0318)	0.1288 (0.0087)
$PLENC_2$	1.0177 (0.0109)	0.0856 (0.0525)	0.0682 (0.0072)
$PLENC_3$	1.0086 (0.0104)	0.0526 (0.0637)	0.0604 (0.0067)
$PLENC_4$	1.0000 (0.0105)	< 0	0.0366 (0.0067)
$PLENC_5$	1.0155 (0.0111)	0.0745 (0.0534)	0.0704 (0.0074)
PPG PCA_1	1.0146 (0.01)	0.1143 (0.0788)	0.0435 (0.0064)
PPG PCA_2	1.0073 (0.0109)	0.0486 (0.0728)	0.0569 (0.0068)
PPG PCA_3	0.9929 (0.0094)	< 0	0.0224 (0.0059)
PPG PCA_4	1.0071 (0.0114)	0.0302 (0.0487)	0.0876 (0.0078)
PPG PCA_5	1.0087 (0.0093)	0.1079 (0.1158)	0.0264 (0.006)
Absence of Notch	1.0016 (0.0097)	0.0145 (0.085)	0.0409 (0.0061)
Position of Notch	1.0049 (0.0121)	0.023 (0.0572)	0.0762 (0.0074)
Position of Peak	0.9972 (0.0099)	< 0	0.0662 (0.0068)
Position of Shoulder	1.0004 (0.0107)	0.0024 (0.0586)	0.0699 (0.0073)
Peak to Peak Time	1.0082 (0.0117)	0.1166 (0.1656)	0.024 (0.0066)
$RPLENC_1$	1.0107 (0.0102)	0.0721 (0.0689)	0.0546 (0.0067)
$RPLENC_2$	1.0125 (0.0107)	0.0779 (0.0672)	0.0554 (0.0067)

Supplementary Table 10: **S-LDSC results on SPINCs, RSPINCs, PLENCs, RPLENCs, PCA GWAS.** We computed the S-LDSC intercept, attenuation ratio and SNP-heritability. Values in parentheses are the standard error of the mean (s.e.m) obtained from S-LDSC.

Method	E[χ^2] All variants	E[χ^2] GWAS catalog Hits
SPINCs (5)	**7.763 (0.003)**	**114.839 (2.830)**
PCA (5)	6.615 (0.002)	60.626 (1.208)
PCA (4)	5.564 (0.002)	58.522 (1.207)
PCA (3)	4.470 (0.002)	55.503 (1.177)
PCA (2)	3.380 (0.002)	52.874 (1.168)
PCA (1)	1.861 (0.001)	16.269 (0.335)

Supplementary Table 11: **SPINCs GWAS power comparison with PCA for spirogram**. We use expected chi-square statistics [1, 2] to compare GWAS power. The number in parentheses for each method indicates the number of latent dimension or embedding. In the case of PCA, we range the number of embeddings from one to five.

Method	E[χ^2] All variants	E[χ^2] GWAS catalog Hits
PLENCs	**5.651 (0.001)**	**15.805 (0.449)**
PCA (5)	5.409 (0.001)	11.755 (0.329)
PCA (4)	4.354 (0.001)	10.109 (0.301)
PCA (3)	3.201 (0.001)	6.159 (0.149)
PCA (2)	2.173 (0.001)	4.692 (0.133)
PCA (1)	1.083 (0.001)	1.831 (0.066)

Supplementary Table 12: **PLENCs GWAS power comparison with PCA for PPG**. We use expected chi-square statistics [1, 2] to compare GWAS power. The number in parentheses for each method indicates the number of latent dimension or embedding. In the case of PCA, we range the number of embeddings from one to five.

Method (# traits)	Sample size	Total	Known (%)	Novel (%)
GWAS Catalog + Shrine 2023	-	1104	–	–
Shrine 2023	581K	754	–	–
Spirogram EDFs (5)	325K	613	581 (95%)	32 (5%)
Spirogram PCA (5)	325K	412	397 (96%)	15 (4%)
Spirogram cubic spline (5)	325K	435	404 (93%)	31 (7%)
SPINCs (5)	325K	575	510 (89%)	65 (11%)
EDFs+RSPINCs (7)	325K	659	596 (90%)	63 (10%)

Supplementary Table 13: **Comparison of GWAS significant loci including cubic spline coefficients.** For lung function and spirograms, expert-defined features (EDFs) are FEV_1, FVC, FEV_1/FVC, PEF, and $FEF_{25\text{-}75\%}$, and "known" and "novel" is in reference to lung function loci in GWAS Catalog and Shrine, et al., 2023. For cardiovascular function and PPG, EDFs are absence of notch, position of notch, position of peak, position of shoulder, and peak-to-peak time, and "known" and "novel" is in reference to cardiovascular disease loci in GWAS Catalog.

Method (# traits)	Sample size	Total	Known (%)	Novel (%)
GWAS Catalog + SpiroMeta	-	1237	–	–
SpiroMeta	-	29	–	–
Spirogram EDFs (5)	325K	613	529 (86%)	84 (14%)
Spirogram PCA (5)	325K	412	380 (92%)	32 (8%)
Spirogram cubic spline (5)	325K	435	384 (88%)	51 (12%)
SPINCs (5)	325K	575	469 (81%)	106 (18%)
EDFs+RSPINCs (7)	325K	659	540 (82%)	119 (18%)

Supplementary Table 14: **Comparison of GWAS significant loci using SpiroMeta as baseline.** For lung function and spirograms, expert-defined features (EDFs) are FEV_1, FVC, FEV_1/FVC, PEF, and $FEF_{25\text{-}75\%}$, and “known” and “novel” is in reference to lung function loci in GWAS Catalog and SpiroMeta [4].

Chrom	Gene	P	cond. P	Variant ID	Range	Source
1	DNM3	2.30E-11	2.28E-10	1:172312769_G_A	171809235-172463995	G
2	ATP6V1E2	3.00E-09	3.60E-09	2:46737464_G_C	46631429-47022818	I
2	COL4A3	6.90E-09	9.39E-09	2:228161170_T_C	228118156-228345413	I
3	PLXND1	2.50E-11	2.25E-10	3:129274936_A_G	129056595-129956119	I
3	RBMS3	1.00E-08	2.23E-08	3:30304919_GT_G	30270159-30484211	I
4	AC097375.2	2.60E-09	9.07E-08	4:152949327_T_TA	152782878-153100189	I
4	BMP3	1.30E-16	3.44E-16	4:81952637_T_A	81208992-82124698	I
4	UNC5C	2.00E-09	3.97E-09	4:96573857_C_T	96451871-96666203	I
5	AC079465.1	1.80E-11	9.27E-11	5:112739130_G_A	112532935-113118026	G
5	MCC	1.80E-11	9.27E-11	5:112739130_G_A	112532935-113118026	G
5	CAMK2A	2.20E-14	3.54E-13	5:149625611_A_T	149565508-149665683	I
7	FOXK1	4.00E-09	1.16E-08	7:4692566_A_G	4676340-4746488	I
8	HNF4G	3.00E-09	2.50E-09	8:76362337_G_C	76295984-77021947	I
8	NRG1	9.80E-09	1.28E-07	8:32963969_G_C	32642704-33263514	I
10	AKR1C1	8.80E-09	2.21E-08	10:4961278_T_C	4829609-5108821	I
10	BICC1	1.20E-09	4.13E-10	10:60343348_G_C	60169970-60396362	I
10	VTI1A	4.10E-09	7.83E-09	10:114606290_C_T	114523887-114731845	I
11	CHRDL2	4.60E-22	2.68E-22	11:74427921_C_T	74376844-74838572	I
11	CYB561A3	2.10E-14	1.03E-14	11:61126858_C_T	60838260-61282934	G
11	MRPL23	7.40E-09	4.22E-08	11:2019174_C_T	1874072-2041831	I
15	EMC7	6.00E-09	3.60E-09	15:34379605_C_T	34166481-34424891	G
15	SEMA6D	1.50E-10	6.63E-11	15:47741212_T_G	47649593-47991515	G
17	KCNJ16	1.10E-17	3.24E-17	17:67962340_C_G	67544154-68024377	I
19	FCHO1	8.00E-09	2.56E-08	19:17862267_TC_T	17818037-17895874	I
22	TRIOBP	7.70E-13	2.47E-11	22:38176979_T_G	37977713-38449820	I

Supplementary Table 15: **Potentially novel significant GWAS loci from SPINCs.** Using a stricter P-value of 1×10^{-8}, only displaying loci not found in Shrine et al 2023, GWAS catalog lung function search, or our own GWAS on EDFs. The P-values are from BOLT-LMM GWAS and the conditional P-values ("cond. P") are obtained by conditional analysis on previously known variants as implemented in GCTA-COJO. The closest genes are assigned to each variant. Variant IDs are in the form "chromosome:position_reference_alternate" using GRCh37 reference. In the "Source" column, "G" implies genotyped variants and "I" implies imputed variants.

Chrom	Gene	P	cond. P	Variant ID	Range	Source
1	HHIPL2	1.40E-10	1.05E-09	1:222548602_T_C	222236497-222560502	I
2	LPIN1	2.20E-09	8.13E-09	2:12065180_A_G	12044820-12145188	I
2	PRKCE	1.10E-09	7.43E-10	2:46218502_A_ATT	46165972-46285524	I
2	TMEM247	7.70E-10	4.06E-10	2:46692974_C_CT	46583593-46870757	I
3	H1-8	9.00E-11	6.84E-10	3:129263140_A_G	129056595-129956119	G
4	BMP3	2.20E-28	7.16E-28	4:81952637_T_A	81208992-82124698	I
4	OCIAD1	1.70E-09	5.92E-08	4:48810179_G_A	48342682-53065669	I
5	AC010451.3	1.50E-09	5.27E-08	5:4962498_C_T	4940255-5067870	I
5	AC027343.2	1.00E-08	5.23E-08	5:7158442_G_A	7143293-7371420	I
5	MIR4458HG	2.70E-09	1.98E-09	5:8531288_C_G	8495149-8584403	I
6	ALDH8A1	7.50E-10	9.39E-10	6:135117710_TA_T	135022253-135165945	I
6	TBX18	3.00E-12	1.83E-12	6:85211448_T_C	85134017-85581296	I
7	AC019117.4	1.70E-09	2.02E-08	7:17441082_C_G	17169922-17569101	I
7	FERD3L	1.00E-11	1.58E-11	7:19446881_GT_G	19223257-19630474	I
8	LINC02855	1.30E-12	5.45E-14	8:122668595_T_G	122625186-122792872	I
8	MRPS28	8.10E-09	3.19E-08	8:80756803_A_G	80605017-81070612	I
8	ZNF703	9.70E-09	2.63E-08	8:37532984_A_G	37408632-37658001	G
10	AKR1C1	6.70E-10	1.14E-08	10:4985193_T_C	4829609-5108821	I
10	SLC16A9	2.30E-09	5.46E-10	10:61320597_G_A	61320597-61380392	I
11	GRM5	1.00E-11	3.33E-12	11:88486055_A_G	88329190-88952464	I
11	NAV2	1.40E-18	2.37E-17	11:19973306_C_G	19965487-20019667	I
11	QSER1	6.00E-13	8.42E-13	11:32956492_C_T	32385925-33241651	G
11	XRRA1	2.50E-28	4.13E-27	11:74628743_C_T	74413843-74838572	I
11	YAP1	1.70E-09	2.24E-09	11:102002913_C_T	101761385-102157900	I
13	LINC01069	5.50E-09	1.06E-08	13:78651299_G_A	78125293-78807836	I
14	FLRT2	6.60E-10	1.23E-08	14:86646016_T_G	86643782-86646282	I
15	MCTP2	7.80E-10	4.86E-09	15:94357066_C_T	94273406-94508663	I
15	SEMA6D	1.70E-17	2.27E-17	15:47734845_A_G	47649593-47991515	I
20	PTPN1	5.00E-09	4.24E-10	20:49096493_A_T	48986299-49238073	I
22	TRIOBP	4.10E-18	1.99E-16	22:38176979_T_G	37977713-39285885	I

Supplementary Table 16: **Potentially novel significant GWAS loci from RSPINCs.** Using a stricter P-value of 1×10^{-8}, only displaying loci not found in Shrine et al 2023, GWAS catalog lung function search, or our own GWAS on EDFs. The P-values are from BOLT-LMM GWAS and the conditional P-values ("cond. P") are obtained by conditional analysis on previously known variants as implemented in GCTA-COJO. The closest genes are assigned to each variant. Variant IDs are in the form "chromosome:position_reference_alternate" using GRCh37 reference. In the "Source" column, "G" implies genotyped variants and "I" implies imputed variants.

Chrom	Gene	P	Variant ID	Range	Source
1	AKR1A1	1.50E-12	1:46026397_CT_C	44973546-46891925	I
1	NOS1AP	6.20E-09	1:162161339_C_G	162014632-162265976	I
2	EFEMP1	4.30E-09	2:56095994_C_G	55666224-56257941	I
3	GLYCTK	1.10E-11	3:52333671_C_G	52214640-53553745	I
3	ITGA9	1.20E-09	3:37596805_C_G	37520793-37663628	I
3	LINC02029	1.10E-10	3:156795414_G_T	156791268-156848024	I
3	RSRC1	2.50E-10	3:158173507_G_A	157582078-158540961	I
4	PPARGC1A	1.40E-09	4:23951018_C_G	23926728-24114572	I
7	MKLN1	3.60E-09	7:130973495_C_T	130946690-131213850	I
10	NOC3L	6.20E-09	10:96122543_A_G	95971321-96993955	G
11	FADS1	2.60E-17	11:61569830_C_T	61523300-61678754	G
11	FADS2	2.60E-17	11:61569830_C_T	61523300-61678754	G
13	FGF9	3.60E-11	13:22861921_A_G	22853646-22911560	I
16	CDH13	3.00E-09	16:82750051_A_G	82679422-82924972	I
16	CNOT1	4.60E-09	16:58566304_G_A	58525312-58866367	G
16	HNRNPA1P48	8.40E-09	16:51578359_C_G	51451739-52103288	I
16	TEKT5	1.60E-10	16:10740982_G_C	10695121-10810459	I
18	FHOD3	1.60E-13	18:34289285_G_T	34185526-34942005	I
19	CILP2	1.90E-10	19:19649748_G_C	19087498-19865077	I
20	GNAS	3.40E-09	20:57466093_G_T	57221133-57544177	I

Supplementary Table 17: **Potentially novel significant GWAS loci from PLENCs.** All loci not found in GWAS Catalog cardiovascular disease search. The closest genes are assigned to each variant. Variant IDs are in the form "chromosome:position_reference_alternate" using GRCh37 reference. In the "Source" column, "G" implies genotyped variants and "I" implies imputed variants. The P-values are from BOLT-LMM GWAS

Method	AUC-ROC	AUC-PR	Top decile prevalence	Pearson R
Ratio (1)	0.534 (0.530–0.538)	0.152 (0.149–0.155)	0.160 (0.153–0.167)	0.039 (0.034–0.045)
EDFs (5)	0.540 (0.534–0.545)	0.157 (0.154–0.160)	0.172 (0.165–0.179)	0.049 (0.042–0.055)
Raw PC (5)	0.524 (0.520–0.530)	0.149 (0.146–0.152)	0.153 (0.146–0.161)	0.030 (0.024–0.035)
Cubic Spline (5)	0.529 (0.524–0.533)	0.151 (0.148–0.154)	0.162 (0.155–0.169)	0.035 (0.029–0.041)
EDFs+RSPINCs (7)	0.549 (0.544–0.553)*	0.161 (0.158–0.165)*	**0.182 (0.174–0.189)***	0.060 (0.054–0.066)*
SPINCs (5)	**0.553 (0.549–0.557)***	**0.163 (0.160–0.167)***	**0.182 (0.175–0.189)***	**0.065 (0.058–0.070)***

Supplementary Table 18: **Asthma PRS performance in UK Biobank**. "Ratio" = FEV_1/FVC, "Manual" = {FVC, FEV_1, PEF, $FEF_{25\text{-}75\%}$, FEV_1/FVC}. * statistically significant improvement over "EDFs (5)" with paired bootstrapping with 95% confidence.

Method	AUC-ROC	AUC-PR	Top decile prevalence	Pearson R
Ratio (1)	0.542 (0.536–0.548)	0.073 (0.071–0.076)	0.080 (0.075–0.085)	0.036 (0.031–0.041)
EDFs (5)	0.547 (0.541–0.554)	0.075 (0.072–0.077)	0.083 (0.077–0.088)	0.041 (0.036–0.047)
Raw PC (5)	0.525 (0.518–0.532)	0.069 (0.067–0.071)	0.074 (0.069–0.079)	0.022 (0.016–0.028)
Cubic Spline (5)	0.528 (0.522–0.536)	0.071 (0.068–0.073)	0.077 (0.071–0.082)	0.025 (0.020–0.031)
EDFs+RSPINCs (7)	**0.551 (0.544–0.557)***	**0.076 (0.073–0.078)***	0.084 (0.078–0.089)	**0.044 (0.039–0.050)***
SPINCs (5)	0.549 (0.543–0.556)	**0.076 (0.073–0.079)**	**0.086 (0.080–0.091)**	**0.044 (0.038–0.050)**

Supplementary Table 19: **COPD PRS performance in UK Biobank**. "Ratio" = FEV_1/FVC, "EDFs" = {FVC, FEV_1, PEF, $FEF_{25\text{-}75\%}$, FEV_1/FVC}. * indicates statistically significant improvement over "EDFs (5)" with paired bootstrapping with 95% confidence.

PRS pheno	Asthma PRS weight	COPD PRS weight
$SPINC_1$	0.0217	0.0057
$SPINC_2$	0.0581	0.0213
$SPINC_3$	-0.0402	-0.0285
$SPINC_4$	-0.0655	0.0073
$SPINC_5$	-0.1650	-0.0188
FEV_1	0.0049	0.0188
FVC	-0.0227	-0.0374
PEF	0.0022	0.0001
FEV_1/FVC	-0.4720	-0.3541
$FEF_{25\text{-}75\%}$	-0.0471	-0.0090

Supplementary Table 20: **SPINCs and EDFs PRS weights for pulmonary traits.**

	Method	AUC-ROC	AUC-PR	Top decile prevalence	Pearson R
Non-Hispanic White	EDFs	0.586	0.604	0.650	0.154
	EDFs + RSPINCs	0.589*	0.605	0.656	0.158*
	SPINCs	**0.622***	**0.635***	**0.715***	**0.212***
African American	EDFs	0.538	0.358	0.360	0.064
	EDFs + RSPINCs	0.536	0.356	0.358	0.062
	SPINCs	**0.559***	**0.372**	**0.374**	**0.096***

Supplementary Table 21: **COPDGene COPD PRS performance**. Bold numbers are the highest in the same category. * indicates statistically significant improvement over EDFs with paired bootstrapping $p < 0.05$. EDFs: FEV_1, FVC, FEV_1/FVC, PEF, and $FEF_{25\text{-}75\%}$.

PRS pheno	HTN PRS weight	SBP PRS weight
$PLENC_1$	0.1326	15.2648
$PLENC_2$	-0.0059	-0.5420
$PLENC_3$	0.0895	2.8647
$PLENC_4$	0.1949	21.6961
$PLENC_5$	-0.0411	-14.0404
Absence of notch	0.3176	18.6592
Position of notch	0.0004	-0.1606
Position of peak	0.0370	4.7233
Position of shoulder	-0.0197	-3.4335
Peak-to-peak time	-0.0012	0.0220

Supplementary Table 22: **PLENCs and EDFs PRS weights for cardiovascular traits.**

Method	AUC-ROC	AUC-PR	Top decile prevalence	Pearson R
EDFs (5)	0.5167 (0.5143-0.5186)	0.4051 (0.4025-0.4073)	0.4162 (0.4099-0.4216)	0.0291 (0.0253-0.0322)
Raw PCA (5)	0.5180 (0.5159-0.5200)	0.4068 (0.4042-0.4094)	0.4222 (0.4163-0.4275)	0.0314 (0.0276-0.0346)
EDFs + RPLENCs (7)	0.5177 (0.5154-0.5195)	0.4056 (0.4029-0.4079)	0.4175 (0.4120-0.4228)	0.0304 (0.0269-0.0336)
PLENCs (5)	**0.5256 (0.5233-0.5275)***	**0.4130 (0.4104-0.4156)***	**0.4296 (0.4235-0.4350)***	**0.0441 (0.0404-0.0476)***

Supplementary Table 23: **PLENCS PRS performance on hypertension (HTN) in UK Biobank.** Bold numbers are the highest in the same category. * indicates statistically significant improvement over EDFs with paired bootstrapping $p < 0.05$.

Method	Pearson R	Spearman R
EDFs (5)	0.0309 (0.0273-0.0348)	0.0303 (0.0265-0.0340)
Raw PCA (5)	0.0394 (0.0361-0.0426)	0.0433 (0.0397-0.0466)
EDFs + RPLENCs (7)	0.0421 (0.0388-0.0460)	0.0421 (0.0387-0.0459)
PLENCs (5)	**0.0603 (0.0561-0.0644)***	**0.0592 (0.0548-0.0631)***

Supplementary Table 24: **PLENCS PRS performance on systolic blood pressure (SBP) in UK Biobank.** Bold numbers are the highest in the same category. * indicates statistically significant improvement over EDFs with paired bootstrapping $p < 0.05$.

SPINC	ID	Description	Beta	R	P-value	SE
$SPINC_3$	continuous-3063-both_sexes-irnt	FEV1	3.014	0.452	< 5.00e-300	0.009
$SPINC_2$	phecode-593-both_sexes	Hematuria	-0.736	-0.444	<5.00e-300	0.002
$SPINC_2$	phecode-695.4-both_sexes	Lupus	0.714	0.431	<5.00e-300	0.002
$SPINC_2$	categorical-20004-both_sexes-1228	Thyroid radioablation therapy	0.716	0.432	<5.00e-300	0.002
$SPINC_3$	continuous-20150-both_sexes-irnt	FEV1, best measure	3.051	0.458	<5.00e-300	0.009
$SPINC_3$	continuous-20154-both_sexes-irnt	FEV1 % predicted	3.409	0.511	<5.00e-300	0.009
$SPINC_2$	categorical-20086-both_sexes-8	Gluten-free diet	0.819	0.494	<5.00e-300	0.002
$SPINC_2$	categorical-41245-both_sexes-1860	Urology consultant	-0.686	-0.414	<5.00e-300	0.002
$SPINC_2$	categorical-6144-both_sexes-3	Never eat wheat	0.879	0.530	<5.00e-300	0.002
$SPINC_2$	phecode-242.1-both_sexes	Graves' disease	0.672	0.405	<5.00e-300	0.002
$SPINC_2$	categorical-1448-both_sexes-4	"Other" bread type	0.826	0.498	<5.00e-300	0.002
$SPINC_2$	continuous-FEV1FVC-both_sexes-irnt	FEV1/FVC ratio	-1.329	-0.802	<5.00e-300	0.002
$SPINC_2$	categorical-20002-both_sexes-1371	Sarcoidosis	0.774	0.467	<5.00e-300	0.002
$SPINC_2$	continuous-3064-both_sexes-irnt	PEF	-0.830	-0.500	<5.00e-300	0.002
$SPINC_2$	icd10-E05-both_sexes	Thyrotoxicosis	0.676	0.408	<5.00e-300	0.002

Supplementary Table 25: **Top associations of SPINCs PRSs with UK Biobank phenotype PRSs.** The PRS of each SPINCs coordinate was compared to phenotype PRSs generated from GWAS summary statistics from the Pan-UKBB consortium. The two-sided P-values of Pearson correlations were computed using the exact distribution of Pearson R under the null hypothesis with independent normal distributions, as implemented in SciPy. Results shown are limited to those with $|R| \geq 0.4$. Full results are available in Supplementary Table 26. R, Pearson R; SE, standard error; FEV1, forced expiratory volume in 1 second; FVC, forced vital capacity; PEF, peak expiratory flow.

See the attached Excel table.

Supplementary Table 26: **All associations of SPINCs PRSs with UK Biobank phenotype PRSs.** Same as Supplementary Table 25, full results.

RSPINC	ID	Description	Beta	R	P-value	SE
$RSPINC_1$	phecode-593-both_sexes	Hematuria	1.007	0.439	<5.00e-300	0.003
$RSPINC_1$	phecode-695.4-both_sexes	Lupus	-1.019	-0.444	<5.00e-300	0.003
$RSPINC_1$	phecode-695.42-both_sexes	Systemic lupus erythematosus	-0.975	-0.425	<5.00e-300	0.003
$RSPINC_1$	icd10-M32-both_sexes	Systemic lupus erythematosus	-0.975	-0.425	<5.00e-300	0.003
$RSPINC_1$	icd10-E05-both_sexes	Thyrotoxicosis	-0.962	-0.419	<5.00e-300	0.003
$RSPINC_1$	phecode-242-both_sexes	Thyrotoxicosis	-0.927	-0.404	<5.00e-300	0.003
$RSPINC_1$	categorical-41245-both_sexes-1860	Urology consultant	0.987	0.430	<5.00e-300	0.003
$RSPINC_1$	categorical-6144-both_sexes-3	Never eat wheat	-1.154	-0.503	<5.00e-300	0.003
$RSPINC_2$	categorical-20533-both_sexes-20533	Trouble falling asleep	25.894	0.560	<5.00e-300	0.058
$RSPINC_1$	categorical-20004-both_sexes-1228	Thyroid radioablation therapy	-0.955	-0.416	<5.00e-300	0.003
$RSPINC_1$	categorical-41200-both_sexes-M459	Unspecified diagnostic endoscopic bladder exam	0.946	0.412	<5.00e-300	0.003
$RSPINC_1$	categorical-41200-both_sexes-W365	Diagnostic extraction of bone marrow NEC	-0.980	-0.427	<5.00e-300	0.003
$RSPINC_1$	categorical-20086-both_sexes-8	Gluten-free diet	-1.089	-0.475	<5.00e-300	0.003
$RSPINC_1$	categorical-1448-both_sexes-4	"Other" bread type	-1.116	-0.486	<5.00e-300	0.003
$RSPINC_1$	categorical-20002-both_sexes-1371	Sarcoidosis	-1.054	-0.459	<5.00e-300	0.003
$RSPINC_1$	continuous-3064-both_sexes-irnt	PEF	1.166	0.508	<5.00e-300	0.003
$RSPINC_1$	continuous-FEV1FVC-both_sexes-irnt	FEV1/FVC ratio	1.218	0.530	<5.00e-300	0.003

Supplementary Table 27: **Top associations of RSPINCs PRSs with UK Biobank phenotype PRSs.** The PRS of each RSPINCs coordinate was compared to phenotype PRSs generated from GWAS summary statistics from the Pan-UKBB consortium. The two-sided P-values of Pearson correlations were computed using the exact distribution of Pearson R under the null hypothesis with independent normal distributions, as implemented in SciPy. Results shown are limited to those with $|R| \geq 0.4$. Full results are available in Supplementary Table 26. R, Pearson R; SE, standard error; NEC, necrosis; PEF, peak expiratory flow; FEV1, forced expiratory volume in 1 second; FVC, forced vital capacity.

See the attached Excel table.

Supplementary Table 28: **All associations of RSPINCs PRSs with UK Biobank phenotype PRSs.** Same as Supplementary Table 27, full results.

PLENCs	ID	Description	Beta	R	P-value	SE
$PLENC_2$	continuous-12336-both_sexes-irnt	Ventricular rate	-2.909	-0.438	$<$5.00e-300	0.009
$PLENC_2$	continuous-95-both_sexes-irnt	Pulse rate (during blood-pressure measurement)	-3.348	-0.504	$<$5.00e-300	0.009
$PLENC_1$	continuous-4194-both_sexes-irnt	Pulse rate	-4.504	-0.678	$<$5.00e-300	0.007
$PLENC_1$	continuous-102-both_sexes-irnt	Pulse rate, automated reading	-3.667	-0.552	$<$5.00e-300	0.008
$PLENC_1$	continuous-5983-both_sexes-irnt	ECG, heart rate	-3.486	-0.525	$<$5.00e-300	0.009

Supplementary Table 29: **Top associations of PLENCs PRSs with UK Biobank phenotype PRSs.** The PRS of each PLENCs coordinate was compared to phenotype PRSs generated from GWAS summary statistics from the Pan-UKBB consortium. The two-sided P-values of Pearson correlations were computed using the exact distribution of Pearson R under the null hypothesis with independent normal distributions, as implemented in SciPy. Results shown are limited to those with $|R| \geq 0.4$. Full results are available in Supplementary Table 30. R, Pearson R; SE, standard error.

See the attached Excel table.

Supplementary Table 30: **All associations of PLENCs PRSs with UK Biobank phenotype PRSs.** Same as Supplementary Table 29, full results.

RPLENCs	ID	Description	Beta	R	P-value	SE
$RPLENC_1$	continuous-4196-both-sexes-irnt	Pulse wave peak to peak time	-6.351038	-0.317940	$<$5.00e-300	0.028705
$RPLENC_1$	categorical-41245-both-sexes-1130	Main speciality of consultant (Clinical haematology)	4.980403	0.249328	$<$5.00e-300	0.029320
$RPLENC_1$	categorical-41244-both-sexes-400	Intended management of patient (No overnight stay)	5.814437	0.291080	$<$5.00e-300	0.028965
$RPLENC_1$	categorical-41246-both-sexes-1220	Treatment speciality of consultant (Clinical haematology)	5.121855	0.256409	$<$5.00e-300	0.029264
$RPLENC_1$	categorical-41200-both-sexes-X362	Venesection	4.955468	0.248080	$<$5.00e-300	0.029330
$RPLENC_1$	categorical-41200-both-sexes-J132	Percutaneous biopsy of lesion of liver NEC	5.167475	0.258693	$<$5.00e-300	0.029245
$RPLENC_1$	icd10-E83-both-sexes	Disorders of mineral metabolism	5.083859	0.254507	$<$5.00e-300	0.029279
$RPLENC_1$	categorical-41246-both-sexes-1620	Treatment speciality of consultant (Hepatology)	4.244111	0.212468	$<$5.00e-300	0.029585
$RPLENC_1$	continuous-21021-both-sexes-irnt	Pulse wave Arterial Stiffness index	6.218144	0.311285	$<$5.00e-300	0.028772
$RPLENC_1$	categorical-41253-both-sexes-70	Inpatient record format (UK HES Inpatients)	4.175468	0.209031	$<$5.00e-300	0.029607
$RPLENC_1$	categorical-41249-both-sexes-1003	Methods of admission to hospital (Elective: Planned)	4.597235	0.230146	$<$5.00e-300	0.029463
$RPLENC_1$	categorical-41247-both-sexes-3000	Patient classification on admission (Regular day attender)	5.798622	0.290289	$<$5.00e-300	0.028972
$RPLENC_1$	phecode-275.1-both-sexes	Disorders of iron metabolism	4.574960	0.229031	$<$5.00e-300	0.029471
$RPLENC_1$	phecode-275-both-sexes	Disorders of mineral metabolism	5.063090	0.253467	$<$5.00e-300	0.029287
$RPLENC_1$	phecode-830-both-sexes	Dislocation	5.081517	0.254390	$<$5.00e-300	0.029280

Supplementary Table 31: **Top associations of RPLENCs PRSs with UK Biobank phenotype PRSs.** The PRS of each RPLENCs coordinate was compared to phenotype PRSs generated from GWAS summary statistics from the Pan-UKBB consortium. The two-sided P-values of Pearson correlations were computed using the exact distribution of Pearson R under the null hypothesis with independent normal distributions, as implemented in SciPy. Results shown are limited to those with $|R| \geq 0.4$. Full results are available in Supplementary Table 32. R, Pearson R; SE, standard error.

See the attached Excel table.

Supplementary Table 32: **All associations of RPLENCs PRSs with UK Biobank phenotype PRSs.** Same as Supplementary Table 31, full results.

Pheno1	Pheno2	Genetic Correlation (rg)
$SPINC_2$	$SPINC_1$	0.1559 (0.0407)
$SPINC_3$	$SPINC_1$	-0.3087 (0.0308)
$SPINC_3$	$SPINC_2$	0.0364 (0.0264)
$SPINC_4$	$SPINC_1$	0.234 (0.0356)
$SPINC_4$	$SPINC_2$	0.0061 (0.0412)
$SPINC_4$	$SPINC_3$	0.1495 (0.0334)
$SPINC_5$	$SPINC_1$	-0.1951 (0.0381)
$SPINC_5$	$SPINC_2$	0.0811 (0.0333)
$SPINC_5$	$SPINC_3$	0.3177 (0.0494)
$SPINC_5$	$SPINC_4$	0.0306 (0.0444)
FEV_1	$FEF_{25\text{-}75\%}$	0.7521 (0.0439)
FVC	$FEF_{25\text{-}75\%}$	0.369 (0.0286)
FVC	FEV_1	0.8962 (0.0535)
PEF	$FEF_{25\text{-}75\%}$	0.699 (0.0444)
PEF	FEV_1	0.6698 (0.0451)
PEF	FVC	0.4112 (0.0349)
FEV_1/FVC	$FEF_{25\text{-}75\%}$	0.8174 (0.0536)
FEV_1/FVC	FEV_1	0.4279 (0.0297)
FEV_1/FVC	FVC	-0.0613 (0.0256)
FEV_1/FVC	PEF	0.5925 (0.036)
Peak to peak time	Absence of notch	-0.5783 (0.1515)
Position of notch	Absence of notch	-0.0413 (0.0682)
Position of notch	Peak to peak time	0.0389 (0.0972)
Position of peak	Absence of notch	0.7017 (0.0809)
Position of peak	Peak to peak time	-0.9171 (0.1458)
Position of peak	Position of notch	0.0861 (0.0661)
Position of shoulder	Absence of notch	0.6078 (0.0851)
Position of shoulder	Peak to peak time	-0.9501 (0.1391)
Position of shoulder	Position of notch	0.2833 (0.0653)
Position of shoulder	Position of peak	1.0031 (0.0854)
$PLENC_2$	$PLENC_1$	0.2917 (0.0689)
$PLENC_3$	$PLENC_1$	-0.0781 (0.0541)
$PLENC_3$	$PLENC_2$	-0.5335 (0.0958)
$PLENC_4$	$PLENC_1$	0.2868 (0.0874)
$PLENC_4$	$PLENC_2$	-0.1771 (0.1072)
$PLENC_4$	$PLENC_3$	0.2404 (0.094)
$PLENC_5$	$PLENC_1$	0.2755 (0.0687)
$PLENC_5$	$PLENC_2$	0.2941 (0.0815)
$PLENC_5$	$PLENC_3$	-0.0207 (0.0925)
$PLENC_5$	$PLENC_4$	-0.0621 (0.1108)

Supplementary Table 33: **Genetic correlation between SPINCs, PLENCS, and EDFs obtained by LDSC.**

Term	Description	Region P	Gene P	Num regions
GO:0048598	embryonic morphogenesis	2.05e-08	1.02e-18	106
GO:0001501	skeletal system development	6.91e-08	2.66e-15	92
GO:0002009	morphogenesis of an epithelium	5.16e-06	1.78e-13	82
GO:0048568	embryonic organ development	5.20e-06	3.22e-13	81
GO:0060562	epithelial tube morphogenesis	2.01e-05	8.68e-13	67
GO:0035239	tube morphogenesis	2.21e-05	2.67e-14	73
GO:0061138	morphogenesis of a branching epithelium	4.12e-05	2.96e-12	48
GO:0001655	urogenital system development	7.85e-05	7.01e-14	71

Supplementary Table 34: **Strongest term enrichments of the RSPINCs plus EDFs loci.** Enrichments were computed using GREAT with default parameters. The 122 total terms significant at Bonferroni-corrected $P \leq 10^{-4}$ by both the region-based one-sided binomial and gene-based one-sided hypergeometric tests [15] were filtered to those with region fold enrichment ≥ 2.

Supplementary References

1. Loh, P.-R. *et al.* Efficient Bayesian mixed-model analysis increases association power in large cohorts. en. *Nat. Genet.* **47,** 284–290 (Mar. 2015).

2. Loh, P.-R., Kichaev, G., Gazal, S., Schoech, A. P. & Price, A. L. Mixed-model association for biobank-scale datasets. en. *Nat. Genet.* **50,** 906–908 (July 2018).

3. Bulik-Sullivan, B. K. *et al.* LD Score regression distinguishes confounding from polygenicity in genome-wide association studies. en. *Nat. Genet.* **47,** 291–295 (Mar. 2015).

4. Shrine, N. *et al.* Multi-ancestry genome-wide association analyses improve resolution of genes and pathways influencing lung function and chronic obstructive pulmonary disease risk. en. *Nat. Genet.* **55,** 410–422 (Mar. 2023).

5. Sollis, E. *et al.* The NHGRI-EBI GWAS Catalog: knowledgebase and deposition resource. en. *Nucleic Acids Res.* **51,** D977–D985 (Jan. 2023).

6. Hemminki, K., Liu, X., Ji, J., Sundquist, K. & Sundquist, J. Subsequent COPD and lung cancer in patients with autoimmune disease. *European Respiratory Journal* **37,** 463–465 (2011).

7. Shen, T.-C. *et al.* Increased risk of chronic obstructive pulmonary disease in patients with systemic lupus erythematosus: a population-based cohort study. *PLOS ONE* **9,** e91821 (2014).

8. Siafakas, N. M. *et al.* Respiratory Muscle Strength in Hyperthyroidism before and after Treatment. *American Review of Respiratory Disease* **146,** 1025–1029. `https://doi.org/10.1164/ajrccm/146.4.1025` (Oct. 1992).

9. Ludvigsson, J. F., Inghammar, M., Ekberg, M. & Egesten, A. A nationwide cohort study of the risk of chronic obstructive pulmonary disease in coeliac disease. *Journal of Internal Medicine* **271,** 481–489 (2012).

10. Sohn, K., Lee, H. & Yan, X. *Learning Structured Output Representation using Deep Conditional Generative Models* in *Advances in Neural Information Processing Systems* (eds Cortes, C., Lawrence, N., Lee, D., Sugiyama, M. & Garnett, R.) **28** (Curran Associates, Inc., 2015).

11. Higgins, I. *et al.* *beta-VAE: Learning Basic Visual Concepts with a Constrained Variational Framework* in *International Conference on Learning Representations* (2017). `https://openreview.net/forum?id=Sy2fzU9gl`.

12. Kim, H. & Mnih, A. *Disentangling by Factorising* in *Proceedings of the 35th International Conference on Machine Learning* (eds Dy, J. & Krause, A.) **80** (PMLR, Oct. 2018), 2649–2658. https://proceedings.mlr.press/v80/kim18b.html.

13. Kumar, A., Sattigeri, P. & Balakrishnan, A. *Variational Inference of Disentangled Latent Concepts from Unlabeled Observations* in *International Conference on Learning Representations* (2018). https://openreview.net/forum?id=H1kG7GZAW.

14. Locatello, F. *et al. Challenging common assumptions in the unsupervised learning of disentangled representations* in *international conference on machine learning* (2019), 4114–4124.

15. McLean, C. Y. *et al.* GREAT improves functional interpretation of cis-regulatory regions. en. *Nat. Biotechnol.* **28,** 495–501 (May 2010).
