# Supplementary material for: Unsupervised representation learning on high-dimensional clinical data improves genomic discovery and prediction
Source: Nat Genet. 2024 Jul 8;56(8):1604–13. doi: 10.1038/s41588-024-01831-6 (PMC11319202; doi:10.1038/s41588-024-01831-6)
Supplement: Supplementary file 2 — Reporting Summary [file 41588_2024_1831_MOESM2_ESM.pdf]

Corresponding author(s): Farhad Hormozdiari
Last updated by author(s): 01/12/2024

Reporting Summary

Nature Portfolio wishes to improve the reproducibility of the work that we publish. This form provides structure for consistency and transparency in reporting. For further information on Nature Portfolio policies, see our Editorial Policies and the Editorial Policy Checklist.

Statistics

For all statistical analyses, confirm that the following items are present in the figure legend, table legend, main text, or Methods section.

n/a	Confirmed	
☐	☒	The exact sample size (*n*) for each experimental group/condition, given as a discrete number and unit of measurement
☐	☒	A statement on whether measurements were taken from distinct samples or whether the same sample was measured repeatedly
☐	☒	The statistical test(s) used AND whether they are one- or two-sided<br>*Only common tests should be described solely by name; describe more complex techniques in the Methods section.*
☐	☒	A description of all covariates tested
☐	☒	A description of any assumptions or corrections, such as tests of normality and adjustment for multiple comparisons
☐	☒	A full description of the statistical parameters including central tendency (e.g. means) or other basic estimates (e.g. regression coefficient) AND variation (e.g. standard deviation) or associated estimates of uncertainty (e.g. confidence intervals)
☐	☒	For null hypothesis testing, the test statistic (e.g. *F*, *t*, *r*) with confidence intervals, effect sizes, degrees of freedom and *P* value noted<br>*Give P values as exact values whenever suitable.*
☒	☐	For Bayesian analysis, information on the choice of priors and Markov chain Monte Carlo settings
☒	☐	For hierarchical and complex designs, identification of the appropriate level for tests and full reporting of outcomes
☒	☐	Estimates of effect sizes (e.g. Cohen's *d*, Pearson's *r*), indicating how they were calculated

Our web collection on statistics for biologists contains articles on many of the points above.

Software and code

Policy information about availability of computer code

Data collection	No software is used for data collection. We have used available UK Biobank and COPDGene data.

Data analysis	Baseline and BaselineLD annotations: https://data.broadinstitute.org/alkesgroup/ldscore<br>BOLT-LMM (v2.3.6) software: https://data.broadinstitute.org/alkesgroup/bolt-lmm<br>COPDGene study: https://www.ncbi.nlm.nih.gov/projects/gap/cgi-bin/study.cgi?study_id=phs000179.v6.p2<br>GCTA (1.93.3beta) software: https://github.com/jianyangqt/gcta<br>GREAT (v4.0.4) software: http://great.stanford.edu<br>GWAS Catalog: https://www.ebi.ac.uk/gwas/<br>Indiana Biobank study: https://indianabiobank.org/<br>Pan-UK Biobank GWAS: https://pan.ukbb.broadinstitute.org<br>PLINK (v1.9) software: https://www.cog-genomics.org/plink1.9<br>TensorFlow: https://www.tensorflow.org<br>UCSC LiftOver: https://genome.ucsc.edu/cgi-bin/hgLiftOver<br>UK Biobank study: https://www.ukbiobank.ac.uk<br>Michigan Imputation Server https://imputationserver.sph.umich.edu/index.html#!pages/home<br>scikit-learn (v1.0.2): https://github.com/scikit-learn/scikit-learn<br>SciPy (v1.9.3): https://github.com/scipy/scipy

For manuscripts utilizing custom algorithms or software that are central to the research but not yet described in published literature, software must be made available to editors and reviewers. We strongly encourage code deposition in a community repository (e.g. GitHub). See the Nature Portfolio guidelines for submitting code & software for further information.

Data

Policy information about availability of data

All manuscripts must include a data availability statement. This statement should provide the following information, where applicable:

- Accession codes, unique identifiers, or web links for publicly available datasets
- A description of any restrictions on data availability
- For clinical datasets or third party data, please ensure that the statement adheres to our policy

UK Biobank study: https://www.ukbiobank.ac.uk and our access was approved under Application 65275. This research used data generated by the COPDGene study (dbGaP accession phs000179.v6.p2), which was supported by NIH grants U01 HL089856 and U01 HL089897. The EPIC-Norfolk study (DOI 10.22025/2019.10.105.00004) and Indiana Biobank. This research used data generated by the eMERGE III study which was obtained from dbGaP under accession phs001584.v2.p2. GWAS summary statistics for SPINCs, RSPINCs, PLENCs, RPLENCs, and EDFs are freely available on Google Cloud Storage at https://console.cloud.google.com/storage/browser/brain-genomics-public/research/regle.

Research involving human participants, their data, or biological material

Policy information about studies with human participants or human data. See also policy information about sex, gender (identity/presentation), and sexual orientation and race, ethnicity and racism.

Reporting on sex and gender	We did not collect new data for this work. We utilized the sex provided by UK Biobank.
Reporting on race, ethnicity, or other socially relevant groupings	We did not collect new data for this work. We utilized genetically inferred ancestry or reported in the study. UK Bionbank: We used individuals whose genetically inferred ancestry (GIA) is European, COPDGene: “Non-Hispanic White” subset (n=6,576) based on study reported ancestry and “African American” subset (n=3,140), eMERGE III: “White” subset (n=8,288) based on study reported ancestry, EPIC-Norfolk: “White” subset (n=8,288) based on study reported ancestry, and Indiana Biobank: All European GIA.We did not report any race or ethnicity. We did apply our method to different ethnicities to illustrate the capability of our method.
Population characteristics	We did not collect new data for this work. We utilize Age, genotype array, BMI, smoking status that are provided by UK Biobank data for GWAS. For COPDGene, eMERGE III, EPIC-Norfolk, and Indiana Biobank, we evaluate PRS on all samples.
Recruitment	We use UK Biobank sample and we did not perform any recruitment.
Ethics oversight	This study utilize public available data such as UK Biobank.

Note that full information on the approval of the study protocol must also be provided in the manuscript.

Field-specific reporting

Please select the one below that is the best fit for your research. If you are not sure, read the appropriate sections before making your selection.

☒ Life sciences ☐ Behavioural & social sciences ☐ Ecological, evolutionary & environmental sciences

For a reference copy of the document with all sections, see nature.com/documents/nr-reporting-summary-flat.pdf

Life sciences study design

All studies must disclose on these points even when the disclosure is negative.

Sample size	We use all the samples provided by UK Biobank.
Data exclusions	We removed samples with excess heterozygosity, missingness, or putative sex chromosome aneuploidy as defined by Bycroft et al 2018 (https://www.nature.com/articles/s41586-018-0579-z). We further limited to individuals of European genetic ancestry as defined in Alipanahi et al 2021 (https://doi.org/10.1016/j.ajhg.2021.05.004)
Replication	We have used GWAS catalog, previous GWAS (Shrine et al. 2023 Nature Genetics; DOI: https://doi.org/10.1038/s41588-018-0321-7 , Sakornsakolpat et al 2019 Nature Genetics; DOI: 10.1038/s41588-018-0342-2) to define nove loci.
Randomization	We use all the samples provided by UK Biobank.
Blinding	No blinding has been performed in our analysis. We use all the samples provided by UK Biobank.

Reporting for specific materials, systems and methods

We require information from authors about some types of materials, experimental systems and methods used in many studies. Here, indicate whether each material, system or method listed is relevant to your study. If you are not sure if a list item applies to your research, read the appropriate section before selecting a response.

Materials & experimental systems

n/a	Involved in the study	
☒	☐	Antibodies
☒	☐	Eukaryotic cell lines
☒	☐	Palaeontology and archaeology
☒	☐	Animals and other organisms
☒	☐	Clinical data
☒	☐	Dual use research of concern
☒	☐	Plants

Methods

n/a	Involved in the study	
☒	☐	ChIP-seq
☒	☐	Flow cytometry
☒	☐	MRI-based neuroimaging
